# Lipidomic and transcriptomic analysis reveals reallocation of carbon flux from cuticular wax into plastid membrane lipids in a glossy “Newhall” navel orange mutant

**DOI:** 10.1038/s41438-020-0262-z

**Published:** 2020-04-01

**Authors:** Haoliang Wan, Hongbo Liu, Jingyu Zhang, Yi Lyu, Zhuoran Li, Yizhong He, Xiaoliang Zhang, Xiuxin Deng, Yariv Brotman, Alisdair R. Fernie, Yunjiang Cheng, Weiwei Wen

**Affiliations:** 1 Key Laboratory of Horticultural Plant Biology (MOE), College of Horticulture and Forestry Sciences, Huazhong Agricultural University, 430070 Wuhan, China; 2 National Key Laboratory of Crop Genetic Improvement, Huazhong Agricultural University, 430070 Wuhan, China; 3 Key Laboratory for Space Bioscience and Biotechnology, School of Life Sciences, Northwestern Polytechnical University, Youyi Xilu 127, Xi’an, 710072 Shaanxi China; 4 Department of Life Sciences, Ben-Gurion University of the Negev, Beersheba, Israel; 5 Max-Planck-Institute of Molecular Plant Physiology, Am Muehlenberg 1, 14476 Potsdam, Germany

**Keywords:** Metabolism, Natural variation in plants

## Abstract

Both cuticle and membrane lipids play essential roles in quality maintenance and disease resistance in fresh fruits. Many reports have indicated the modification of alternative branch pathways in epicuticular wax mutants; however, the specific alterations concerning lipids have not been clarified thus far. Here, we conducted a comprehensive, time-resolved lipidomic, and transcriptomic analysis on the “Newhall” navel orange (WT) and its glossy mutant (MT) “Gannan No. 1”. The results revealed severely suppressed wax formation accompanied by significantly elevated production of 36-carbon plastid lipids with increasing fruit maturation in MT. Transcriptomics analysis further identified a series of key functional enzymes and transcription factors putatively involved in the biosynthesis pathways of wax and membrane lipids. Moreover, the high accumulation of jasmonic acid (JA) in MT was possibly due to the need to maintain plastid lipid homeostasis, as the expression levels of two significantly upregulated lipases (CsDAD1 and CsDALL2) were positively correlated with plastid lipids and characterized to hydrolyze plastid lipids to increase the JA content. Our results will provide new insights into the molecular mechanisms underlying the natural variation of plant lipids to lay a foundation for the quality improvement of citrus fruit.

## Introduction

Waxing and polishing are key steps in the commercialization of fresh citrus. However, due to its unique chemical properties, the synthetic wax used for this purpose blocks stomatal pores and affects fruit quality. Therefore, the cultivation of fruits with desirable colors, high glossiness, fine internal quality, and strong antibacterial properties is of considerable importance. As the first external barrier of plants that come into contact with the environment, epicuticular wax has been reported to play important roles in water retention, gas exchange^1,2^, and surface glossiness of citrus fruits^3,4^.

In conjunction with the external protection of the cuticle, membrane lipids directly protect plant cells and organelles^5^, and plastid membrane lipids are the principal constituents of plant membrane lipid systems. Unlike phosphatidylcholine (PC) and phosphatidylethanolamine (PE), which are dominant in the plasma membrane, galactoglycerolipids, such as monogalactosyldiacylglycerols (MGDGs) and digalactosyldiacylglycerols (DGDGs), together with a fraction (~10%) of phosphatidylglycerols (PGs), serve as the structural lipids that sustain the delicate skeleton of the plastid membrane^6^. In plants, there are two distinct pathways for galactolipid biosynthesis in the plastid and the ER, which are generally known as the “prokaryotic pathway” and “eukaryotic pathway”, respectively. Owing to the substrate preference of lysophosphatidic acyltransferase, galactolipids generated from the “prokaryotic pathway” tend to carry C16 fatty acids at the sn-2 position, while the “eukaryotic pathway” generates galactolipids with C18 fatty acids at the sn-2 position. Therefore, plants are sometimes classified accordingly as “16:3 plants” or “18:3 plants”.

In plants, all lipid compounds share acyl-CoA pools in the cytosol^7^. However, the carbon distribution between different lipid classes could be rigorous or flexible^8^. Among most of the known *cer* (eceriferum) mutants with varying degrees of reduction in wax levels, there seems to be an increase in the flux of precursors into an alternative branch pathway^9^, and *fatb* mutants, with low levels of wax, showed dramatic changes in the formation rate of both prokaryotic and eukaryotic lipids^10^. Thus, knowledge of the variations in lipid contents will provide deep insight into the defense strategy that plants adopt in terms of lipid metabolic pathways. However, few studies have focused on how lipids are altered in cuticle mutants of the model plant *Arabidopsis*, and in crop species, such as citrus, where the cuticle may be highly correlated with industrial value, the number of studies is even smaller.

The promotion of membrane lipid levels may be an effective route to enhance plant performance. Furthermore, an increase in plastid lipids has some unique advantages. First, increased levels of plastid lipids could contribute to highly curved membrane regions for the photosystems, thereby positively affecting the capture of light energy and its conversion^11,12^. Second, previous reports have demonstrated that enhanced tolerance to cold, salt and drought stress corresponds to a higher proportion of highly unsaturated fatty acyls^13,14^, which is one characteristic of plastid lipids.

Modifications of plastid lipids have been well documented^15^. The phospholipase A1 (PLA1) gene family, which functions in the hydrolyzation of the sn-1 position of glycerol backbones, is known to play critical roles in membrane lipid maintenance and synthesis of signaling mediators, such as jasmonic acid (JA), using hydrolyzed products^16,17^. To date, members from two PLA1 gene families have been reported to be involved in JA biosynthesis, namely, the DEFECTIVE ANTHER DEHISCENCE 1-like (DAD1-Like) gene family, including DAD1 and DONGLE (DGL), and the PLASTID LIPASE (PLIP) gene family, including PLIP2 and PLIP3^18–20^. However, controversy regarding their roles still exists: DGL localizes to lipid droplets rather than to chloroplasts, and DAD1 only shows high expression in the late wound response^18^. Although the lipase-catalyzed reaction is regarded as the rate-limiting step in JA synthesis^21^, little is known about which lipase genes are involved in the formation of endogenous JA from plastid lipids in citrus.

To date, most molecular studies related to lipids in citrus have only focused on the cuticle^3,4,22^, without highlighting the importance of membrane lipids. Based on the previously reported glossy mutant of the “Newhall” navel orange^4^, we performed lipidomic and transcriptomic analysis on both immature and mature stages of this glossy mutant (MT) and the wild-type “Newhall” navel orange (WT) to ascertain the carbon exchanges between wax and plastid lipids. We also attempted to elucidate the possible reasons underlying the enhanced JA levels in MT by the functional characterization of three upregulated lipase genes. This study aimed to improve the understanding of quality maintenance in citrus fruits and provide new insights into the molecular mechanisms underlying the naturally occurring variations of lipid pools in citrus.

## Results

### Wax component contents decreased in MT throughout fruit development

**Fig. 1:**
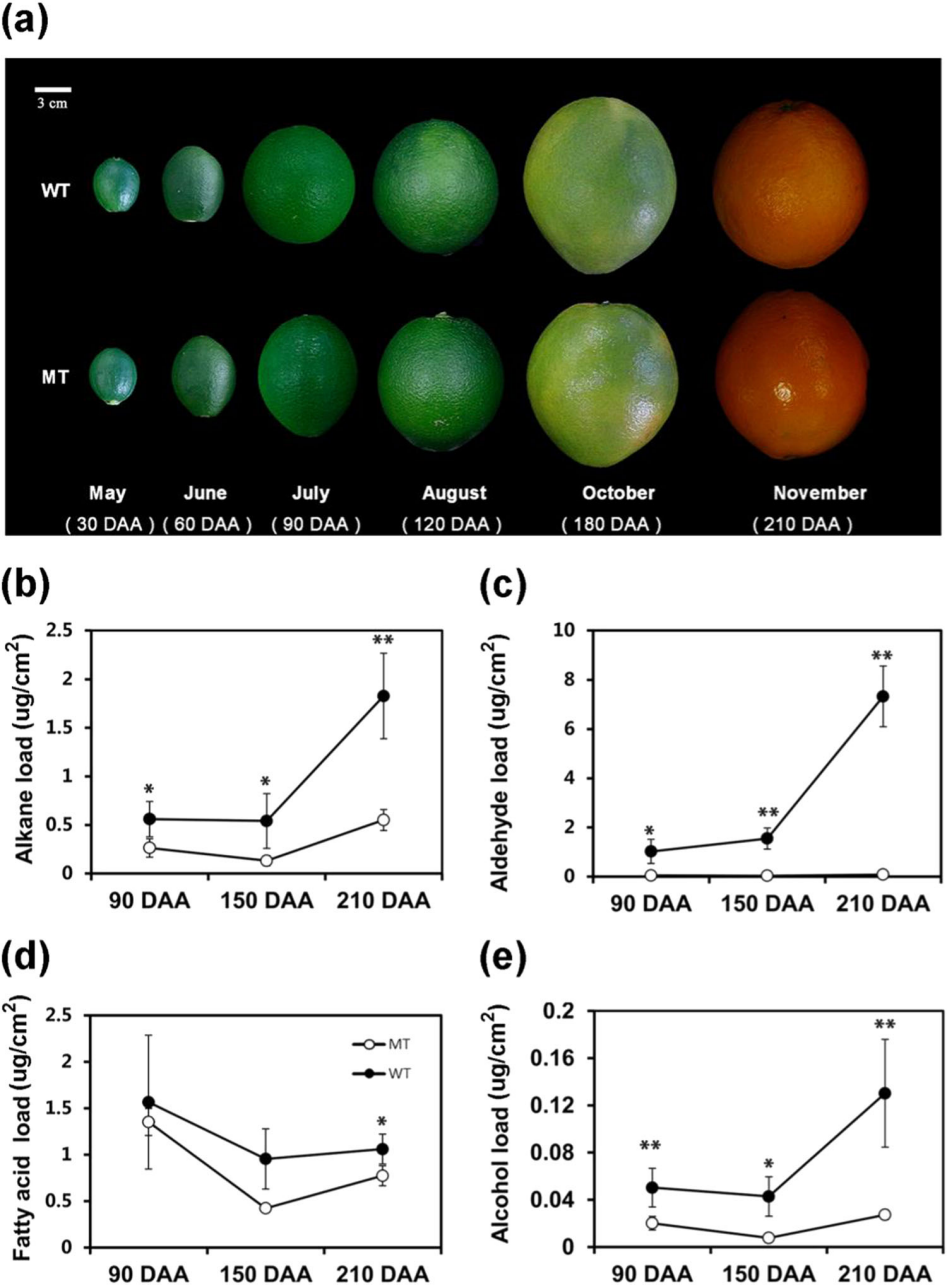
Glossy phenotype and wax synthesis with increasing maturation in MT and WT. **a** Gradual, obvious glossy phenotype of MT compared with that of WT with increasing fruit maturation. DAA days after anthesis, MT mutant type, WT wild type. **b–e** Levels of main wax classes in flavedos of WT and MT in the three stages analyzed. **b** Alkanes, **c** aldehydes, **d** cuticular fatty acids, and **e** primary alcohols. The level of each wax class was the sum of all subclasses. Values are the average of three biological replicates ± standard deviation (SD) (*n* = 3). An asterisk (*) indicates a *t*-test *p* value ≤ 0.05 and double asterisks (**) indicate a *t-*test *p* value ≤ 0.01. More detailed information on the molecular species is available in Supplementary Fig. S1

A glossy phenotype became apparent during the fruit ripening process of MT (Fig. 1a). To further verify how wax contents were altered in developmental stages, we selected three stages: 90 days after anthesis (DAA), 150 DAA and 210 DAA, when fruits started to expand, began to ripen and became fully ripe, respectively. Among the wax components of the “Newhall” navel orange, aldehydes accounted for the largest proportion, followed by that of alkanes, FA, and alcohols^23^. Most aliphatic compounds showed a decreasing, and then sharply increasing, trend, but a continuous accumulation of aldehyde components was observed in both WT and MT (Fig. 1c). All aliphatic compounds in the wax were present in lower concentrations in MT than in WT across the entire developmental process, with slight differences at 90 DAA and increasing differences with increasing fruit maturation. In the case of aldehydes, we first observed a 21-fold change (FC) decrease in MT (0.05 µg/cm^2^) compared with that in WT (1.03 µg/cm^2^) at 90 DAA. At 150 DAA, the FC was 43 (0.04 µg/cm^2^ vs 1.55 µg/cm^2^), and at 210 DAA, the FC peaked at 88 (0.082 µg/cm^2^ vs 7.78 µg/cm^2^). Moreover, the proportion of alkanes in MT was reduced by 70% compared with than in WT, and that of alcohols was reduced by 80% at 210 DAA.

### Glycerol lipids increased in MT

**Fig. 2:**
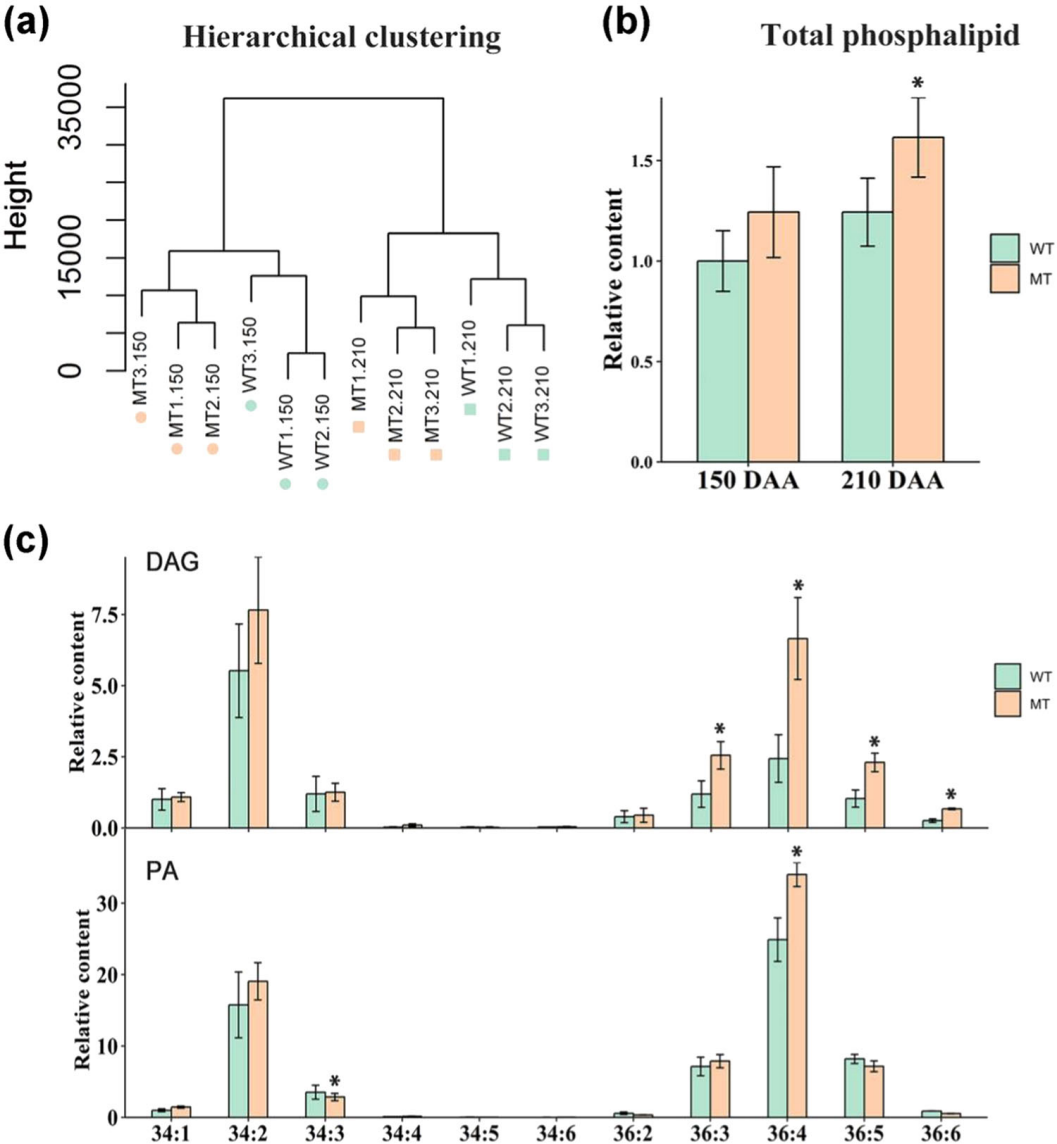
Contents of glycerophospholipids in MT and WT at 150 DAA and 210 DAA. **a** Cluster analysis of all samples for lipid analysis in WT and MT based on average hierarchical clustering. Numbers represent the days after anthesis; WT wild type, MT mutant type. **b** Relative content of total phospholipids observed at 150 and 210 DAA. Corrected areas of all phospholipid classes in each replicate were summed and normalized by the average value number WT at 150 DAA. Bars are the average of three normalized biological replicates ± standard deviation (SD, *n* = 3). **c** Relative contents of two main lipid precursors, PAs and DAGs, at 210 DAA. The corrected area of each subclass was summed and normalized by the average number of 34:1 carbon molecules in DAGs and PAs in WT. Bars are the average of three normalized biological replicates at 210 DAA ± standard deviation (SD, *n* = 3). An asterisk (*) indicates a *t*-test *p* value ≤ 0.05. DAGs diacylglycerols, PAs phosphatidic acids

Wax biosynthesis is one direction of carbon flux from acyl-CoA pools. Thus, the blocking of carbon flux toward wax biosynthesis may have certain feedback effects on the acyl-CoA pools and may subsequently alter membrane lipid metabolism in MT. To verify this hypothesis, we carried out lipidomic profiling on flavedo tissues peeled from fruits at 150 DAA and 210 DAA, when relatively large differences in wax contents were observed (Fig. 1). A total of 13 classes of glycerolipids containing 219 kinds of species were identified and quantified, including 46 TAGs (triacylglycerols), 14 DAGs (diacylglycerols), 20 PCs, 24 PEs, 13 PGs, 14 PIs (phosphatidylinositols), 26 PSs (phosphatidylserines), 12 PAs (phosphatidic acid), 17 MGDGs, 17 DGDGs, 6 LysoPCs (lysophosphatidylcholines), 5 LysoPEs (lysophosphatidylethanolamines), and 5 LysoPGs (lysophosphatidylglycerols) (Supplementary Table S1). As indicated by hierarchical clustering analysis (Fig. 2a), the metabolism of phospholipids in MT showed a distinct pattern from that in WT. Higher lipid accumulations in MT than in WT were observed at both stages according to the relative contents of all lipid classes (Fig. 2b), particularly at 210 DAA. The higher lipid accumulation in MT at 210 DAA was further validated with samples harvested in another year (2017) (Supplementary Fig. S2b and Supplementary Table S2).

**Fig. 3:**
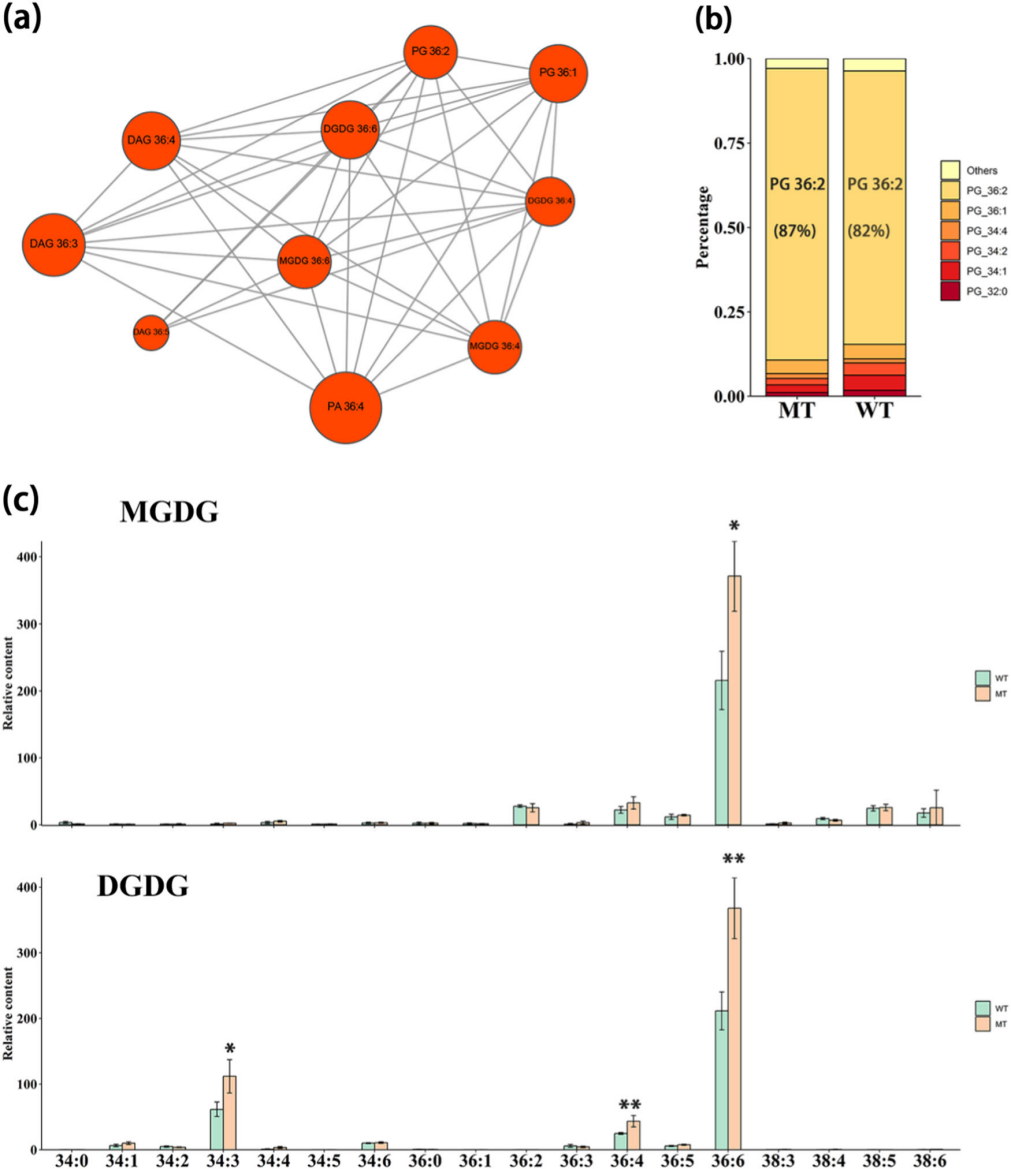
Variations in the main subclasses of plastid lipids at 210 DAA. **a** Network illustrating the highly correlated 36-carbon lipid compounds. Lipid pairs with PCC (Pearson correlation coefficient) values over 0.70 and *p* values < 0.05 are shown. The bubble size indicates the number of correlated metabolites. **b** Proportions of subclasses in the PG class. Subclasses accounting for more than 1% in each class at 210 DAA are displayed in the diagram. Other lipids are classified into “Others,” and those with higher proportions are marked on the diagram. **c** Relative contents of two main chloroplast lipids, MGDGs and DGDGs, at 210 DAA. The corrected area of each subclass was summed and normalized by the average number of 34:2 and 34:4 carbon molecules in MGDGs and DGDGs in WT, respectively. Bars are the average of three normalized biological replicates at 210 DAA ± standard deviation (SD, *n* = 3). An asterisk (*) indicates a *t*-test *p* value ≤ 0.05 and double asterisks (**) mean *t-*test *p* value ≤ 0.01. MGDGs monogalactosyldiacylglycerols, DGDGs digalactosyldiacylglycerols, PG phosphatidylglycerol

Phosphatidic acids are essential intermediates in the synthesis of diverse glycerolipids. Here, we found a similar trend in the variations of levels and carbon species between PAs and DAGs. For example, 36:4 and 34:2 carbon molecules were coincidently dominant in both lipid classes and were simultaneously enhanced in MT (Fig. 2c), and 36:4 PA showed a strong correlation (Pearson correlation coefficient (PCC) ≥ 0.7, *p* ≤ 0.05) with 36:3,4,5-carbon DAGs (Fig. 3e and Supplementary Table S3). Similar results were obtained in 2017 in which 34:2, 36:3, and 36:4 carbon molecules were shown to account for a larger proportion of both PAs and DAGs, and all of them showed high accumulations in MT (Supplementary Fig. S2c). These results suggested a tight metabolic connection between PAs and DAGs in the flavedo of “Newhall” navel oranges.

Further analysis of DAGs showed that the accumulations of 36 highly unsaturated carbon molecules, including 36:3, 36:4, 36:5, and 36:6, were all significantly (*t*-test *p* value ≤ 0.05) elevated in MT relative to those in WT at 150 DAA and 210 DAA (Fig. 2c). Given that 36-carbon DAG could provide skeletons for the biosynthesis of 36-carbon chloroplast galactolipids, it was interesting to observe that 36:6-carbon MGDG and 36:6-carbon DGDG were significantly increased (*t*-test *p* value ≤ 0.05) in MT, and 36:3,4,5-carbon DAGs showed a strong correlation with 36-carbon plastid lipids, such as 36:6 MGDG, 36:6 DGDG, and 36:2 PG (Fig. 3a and Supplementary Table S3). In addition, the dominant proportion of 36:6 molecules in MGDGs and DGDGs (over 60%) in WT and MT (Fig. 3c) demonstrated that the “Newhall” orange mainly depends on the eukaryotic pathway for galactolipid synthesis, similar to most reported seed plants, indicating that “Newhall” orange is a typical “18:3 plant”. DAGs could also provide skeletons for the biosynthesis of TAGs. However, less obvious changes in the levels of TAG subclasses were observed between WT and MT at both 150 DAA and 210 DAA (Supplementary Fig. S3). Interestingly, 36:2 carbon species were dominant in PGs (Fig. 3b) and were significantly upregulated in MT, also suggesting that the ER is the main source of carbon for PG biosynthesis. Similar results were observed in samples harvested in 2017 (Supplementary Fig. S4). Overall, our data showed that plastid lipids, together with the phospholipid precursors PAs and DAGs, were increased in MT compared with those in WT.

**Fig. 4:**
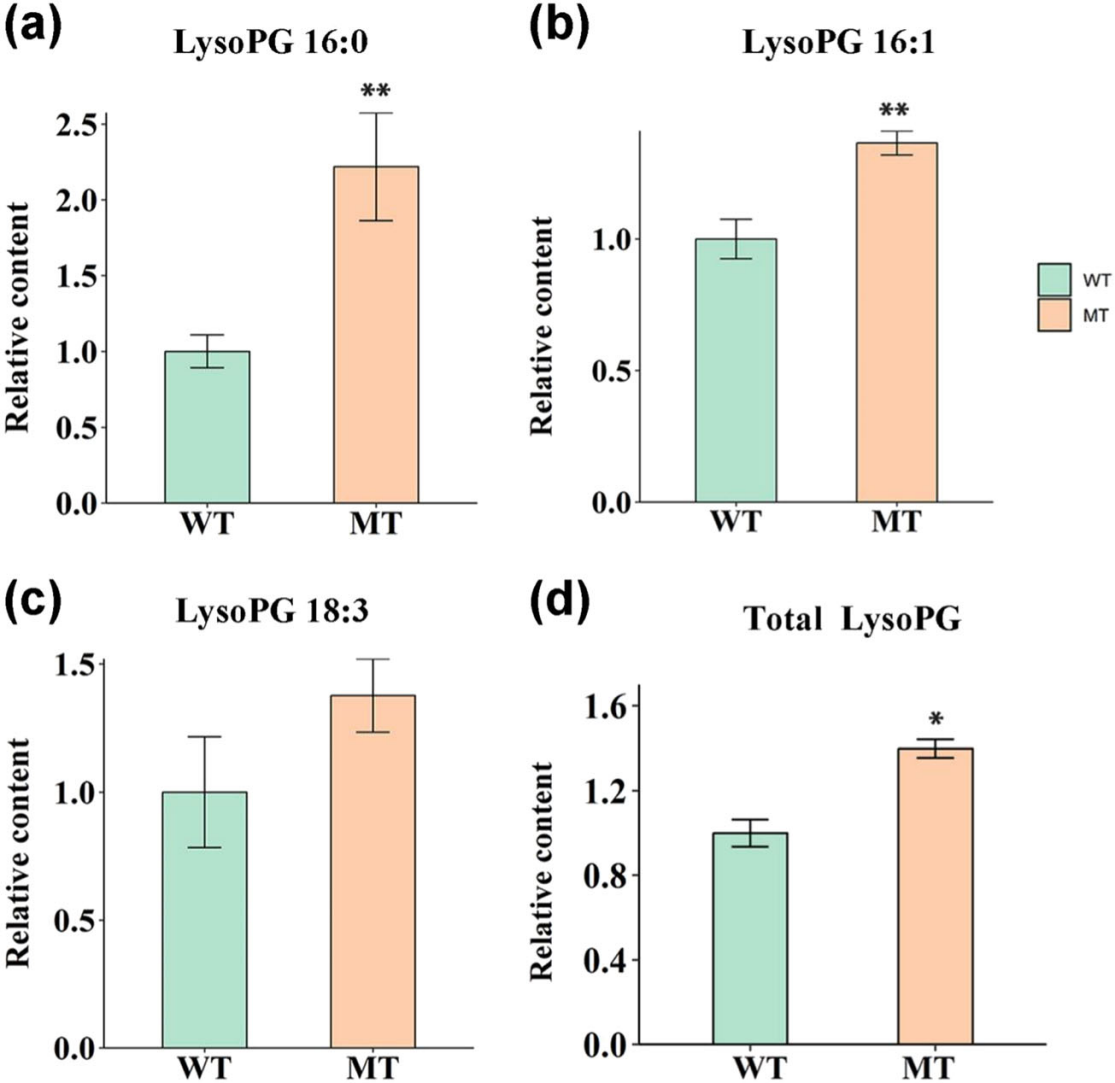
Contents of plastid lipid degradation products, lysoPGs at 210 DAA. **a–c** Relative content of three main lysoPG subclasses in MT compared with that in WT at 210 DAA. **a** LysoPG 16:0; **b** LysoPG 16:1; **c** LysoPG 18:3. **d** Relative content of total lyso PGs in MT compared with WT on 210 DAA. Corrected areas of each lysoPG subclass were summed and normalized by the average number in WT. Bars are the average of three normalized biological replicates ± standard deviation (SD, *n* = 3). An asterisk (*) indicates a *t-*test *p* value ≤ 0.05 and double asterisks (**) indicate a *t-*test *p* value ≤ 0.01

The increase in the three most abundant subclasses of lyso-PGs, the possible hydrolyzation products of glycerophospholipids (Fig. 4a–c) at the mature stage, suggested a possible induction of the phospholipid hydrolyzation pathway in MT. Given that the biosynthesis of JA originates from the degradation of chloroplast lipids, we then tested the JA level at 210 DAA. JA was significantly accumulated (*t*-test *p* value ≤ 0.05) in the flavedo and juice sacs of MT (Supplementary Fig. S5), demonstrating that JA biosynthesis was strongly affected by the altered lipid metabolism in MT in 2014.

### Transcriptomic differences in the flavedo between MT and WT during developmental stages

**Fig. 5:**
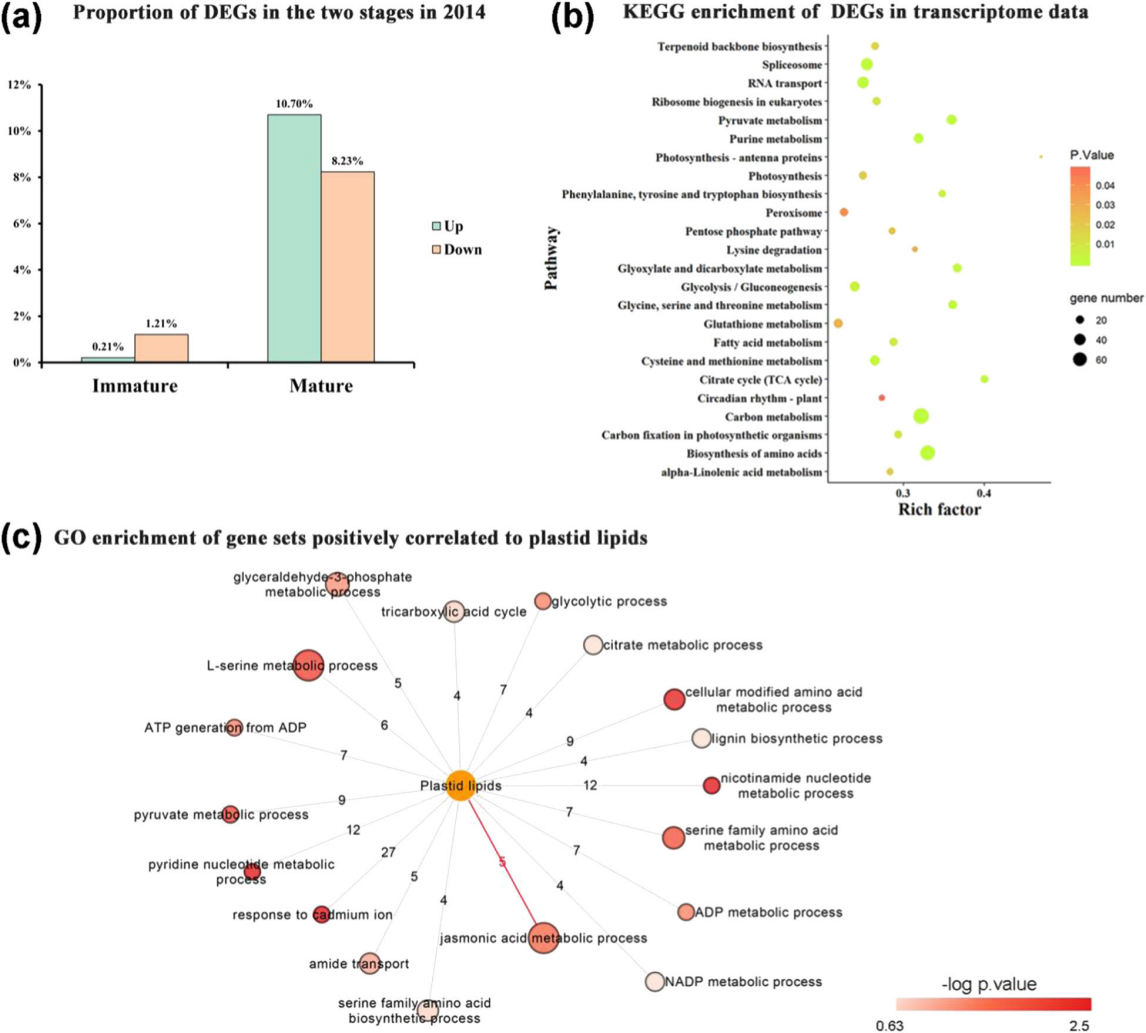
Transcriptomic analysis of WT and MT. **a** Proportions of DEGs in the two developmental stages. The proportions were calculated as the number of DEGs/number of expressed genes in the respective month. **b** The first 20 enriched pathways according to KEGG analysis of the total DEGs characterized in both immature and mature stages. The size of the bubble indicates the input gene number in the pathway, and the color indicates the *p* value. **c** Significantly enriched gene ontology (GO) terms (metabolic process) of genes that positively correlated (PCC ≥ 0.7, *p* ≤ 0.05) with the three main plastid lipids, MGDG 36:6, DGDG 36:6, and PG 36:2. Genes showing a high positive correlation with each of the three plastid lipids were selected, intersected and used for GO enrichment analysis. The color of the bubble indicates enrichment factor, and the color scale indicates the *p* value. The numbers of input genes are indicated on the lines

To further investigate the differences in molecular biological regulation between MT and WT, a transcriptomic analysis was performed on the fruit flavedo harvested at 150 DAA and 210 DAA. Principal component analysis indicated that the differences between the two stages were larger than those between WT and MT (Supplementary Fig. S6a). Thus, we mainly focused on the differentially expressed genes (DEGs) between WT and MT at the same stage. Similar to the gradually larger differences in the lipidomic data, the transcriptomic data revealed that the proportion of DEGs increased with increasing fruit maturation. The number of upregulated genes was smaller at the immature stage and then became similar to that of downregulated genes at the mature stage (Fig. 5a). Similar results were observed in the transcriptomic data at 210 DAA from fruit grown in 2017 (Supplementary Fig. S6b, c). These DEGs were significantly enriched in 24 KEGG pathways. Metabolic pathways such as “photosynthesis”, “photosynthesis-antenna proteins”, and “carbon fixation in photosynthetic organisms”, were closely associated with photosynthesis, while “α-linoleic acid metabolism” and “fatty acid metabolism” were closely related to lipid metabolism (Fig. 5b). To identify pathways involved in plastid lipid metabolism in “Newhall” navel oranges, we generated correlation networks based on lipidomic and transcriptomic data (Supplementary Table S4). Given the dominant proportion and high correlation of 36:6 MGDG and DGDG, and 36:2 PG (Fig. 3), we used the three lipid species to represent the whole plastid lipid profile and focused on overlapping genes that positively correlated with them. A total of 744 genes showed a strong positive correlation with plastid lipids. Among the significantly enriched GO terms of the 744 genes, we found the “JA metabolic process” and “pyruvate metabolism” (Fig. 5c).

To confirm the lipid-related changes at the transcriptional level, we focused on the variations of a compiled list of 93 lipid-related genes among all the up- or down-regulated genes (Supplementary Table S5), which were screened mainly based on a homologous BLAST search against previously released genes in the *Arabidopsis* lipid gene database (http://aralip.plantbiology.msu.edu/pathways/pathways) reported by Li-Beisson et al.^24^. Table 1 presents the lipid genes and some representative transcriptional factors whose expression levels correspond well with the changes in metabolites. Among them, genes that showed a high correlation with MGDG 36:6 were also indicated.

**Table 1:** Summary of key genes involved in the alteration of lipid metabolism pathways

Gene ID	log2 ratio (MT/WT)			PCC (MGDG)	Annotation
	150DAA (2014)	210DAA (2014)	210DAA (2017)		
Wax formation					
Cs2g13450^a^		−1.98		−0.71*	Fatty acyl-ACP thioesterases B
Cs5g28330		−1.36	−1.71	−0.65*	Long-chain acyl-CoA synthetase 2
orange1.1t01720		−0.97	−1.28	−0.86**	Long-chain Acyl-CoA synthetase 1
Cs9g07970		−1.32	−1.26	–	Ketoacyl-CoA synthase 2(a)
Cs6g02360		1.02		0.88**	Ketoacyl-CoA synthase 2(b)
orange1.1t02246^a^		−0.98	−0.78	–	Fatty acyl-CoA reductase/FAR2
Cs5g15350		0.00	−2.67	−0.77**	Fatty acyl-CoA reductase 3
Cs4g02580^a^	−2.51	−1.98	−2.70	–	CER3 protein
Cs3g10550		0.83		0.88**	CER2 protein
orange1.1t02321^a^		−1.42	−2.29	−0.85**	ABCG 32
Cs4g17100		−0.95	−0.73	−0.78**	ABCG 16
Eukaryotic PC turnover					
orange1.1t02241		2.12	0.96	0.71*	Oleate desaturase 2/FAD2
Cs1g12430		1.27	1.94	0.78**	Acyl-CoA desaturase-like/FAD5
Cs6g08600		0.79	0.64	–	Linoleate desaturase/FAD7
Cs7g17090		2.43		0.89**	Glycerol-3-phosphate Acyltransferase 1
Cs5g17310			−0.53	−0.67*	Lysophospholipid acyltransferase
Cs4g10970			1.00	−0.69	1-acylglycerol-3-phosphate acyltransferase 1
Cs1g12600		1.49		–	PL Dalpha 1
Cs7g15610		1.10	0.62	−0.67*	Phospholipase C (Nonspecific) C3
Cs8g20240^a^		−1.50	−1.77	−0.76*	Phosphoinositide-specific phospholipase C4
JA biosynthesis					
Cs2g30480		2.60	2.03	0.80**	Acylhydrolase (DAD1-like)
Cs1g03190		2.06		0.80**	Acylhydrolase (DAD1-like)
Cs5g08030		−0.95		−0.70*	Acylhydrolase (DAD1-like)
orange1.1t04376		1.77		–	LOX2c
Cs2g21000		2.43	0.71	–	HPL1
Cs5g17900		1.23	1.13	–	OPR
Cs5g17920	−1.08	1.52	0.61	–	OPR
Cs3g24230		1.22	0.59	0.85**	Allene oxide synthase
Cs3g25140		3.91	4.80	0.84**	Jasmonic acid carboxyl methyltransferase
Others					
Cs1g13620		1.91	0.56	0.91**	ABCG 40
Cs7g28090		1.29	−0.52	0.87**	ABCG 6
Cs8g15960		−0.78	−1.17	−0.67*	ABCG 3
Cs5g23300		0.90	−0.54	0.91**	Trigalactosyl diacylglycerol 1(a)
Cs6g09960		3.75	3.46	0.82**	Lipid transfer protein type 1
Cs7g06120	−1.19	−2.12		–	SHN ESE3
Cs6g21530		−1.85	−1.30	–	Myb domain protein 16
Cs5g30520		1.96	0.98	0.91**	Pyruvate Kinase
Cs1g17210		−1.31		−0.81**	JASMONATE-ZIM-DOMAIN PROTEIN 1

*PCC* Pearson correlation coefficient. *p value ≤ 0.05, **p value ≤ 0.01

^a^Genes also found in the other two datasets of “Newhall” navel oranges.

### Variations in lipid-related genes across developmental stages

**Fig. 6:**
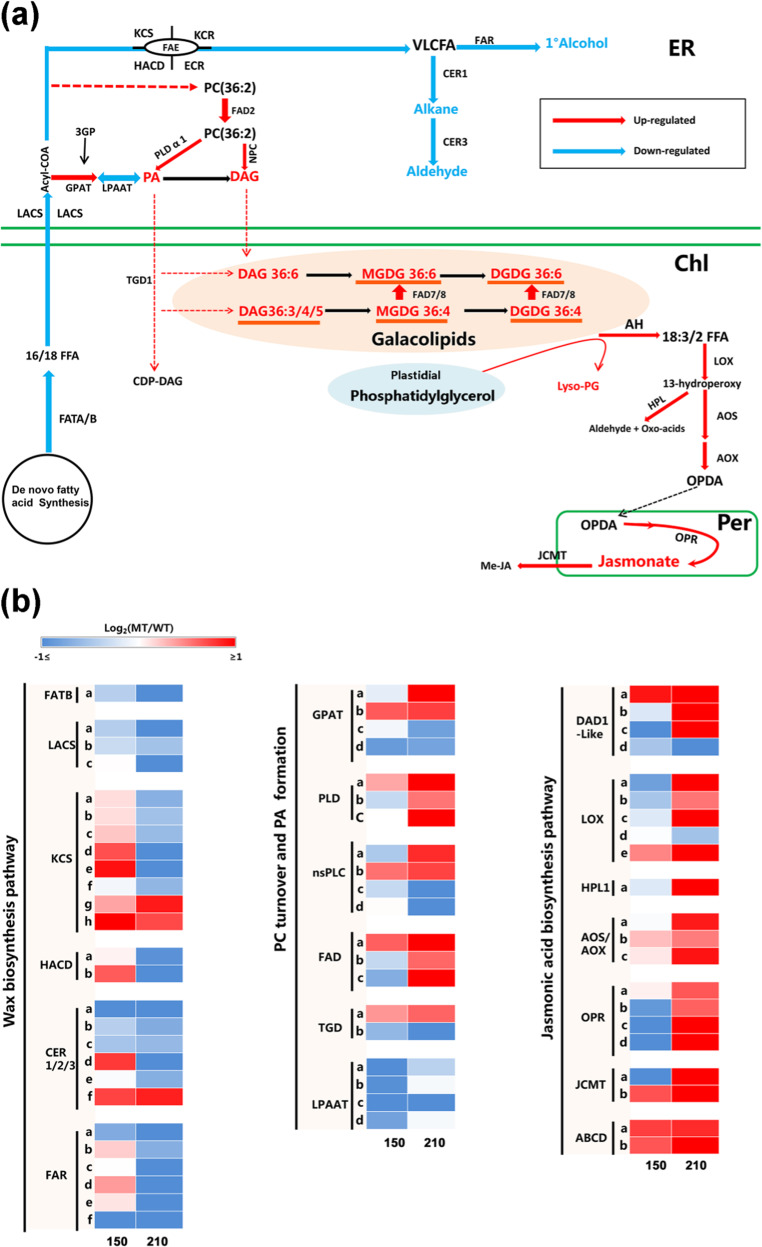
Altered lipid pathways observed in MT at the mature stage. **a** Schematic diagram revealing the main altered lipid pathways at the transcriptional and metabolic levels in MT. The diagram was drawn based on data at the mature stage. Arrows in red or blue indicate transcriptionally up- or down-regulated metabolism pathways; lines in black denote that no genes were found based on our set of standards for transcriptome data; orange lines below phospholipids indicate positive correlation. Characters in red or blue represent up- or down-regulated metabolites. **b** Heatmaps showing log_2_ fold changes of important genes identified for wax, lipid and JA synthesis at the two stages, and the fold change was calculated as MT/WT at the same time point. The genes were identified by a homologous BLAST search against known *Arabidopsis* genes (see “Materials and methods”). More detailed information is available in Supplementary Table S5. Numbers below indicate days after anthesis. ER endoplasmic reticulum, Chl chloroplast, Per peroxisome. FATB fatty acyl-ACP thioesterase B, LACS long-chain acyl-CoA synthetase, KCS ketoacyl-CoA synthase, HACD hydroxyacyl-CoA dehydratase, KCR ketoacyl-CoA reductase, ECR enoyl-CoA reductase, FAR fatty acyl-CoA reductase, CER 1/2/3 eceriferum 1/2/3, GPAT glycerol-3-phosphate acyltransferase, LPAAT 1-acylglycerol-3-phosphate acyltransferase, PLD phospholipase D, nsPLC nonspecific phospholipase C, FAD fatty acid desaturase, AH fatty acyl omega-hydroxylase, LOX lipoxygenase, HPL hydroperoxide lyase, AOS allene oxide synthase, AOX allene oxide cyclase, OPR oxo-phytodienoic acid reductase, JCMT jasmonic acid carboxyl methyltransferase, TGD trigalactosyl diacylglycerol, ABCD ATP-binding cassette acyl transporter D, PC phosphatidylcholine, PG phosphatidylglycerol, DAG diacylglycerol, PA phosphatidic acid, MGDG monogalactosyldiacylglycerol, DGDG digalactosyl diacylglycerol, VLCFA very-long-chain-fatty acid, CDP-DAG CDP-diacylglycerol, FA fatty acid, OPDA oxo-phytodienoic acid, Me-JA methyl jasmonate

Based on the results of the lipidomic and transcriptomic analysis, a schematic diagram was generated to reveal the most significantly altered lipid pathways in MT compared with those in WT (Fig. 6a). Consistent with the gradual decrease in the content of aliphatic wax compounds in MT, the expression levels of gene families involved in wax biosynthesis pathways also displayed a constant decreasing trend in MT (Fig. 6b). However, a number of them showed a high correlation with MGDG 36:6. Fatty acyl-ACP thioesterase B (FATB), LACS1 and LACS2 (long-chain acyl-CoA synthetase), which are responsible for providing precursors for wax synthesis, exhibited downregulated expression in MT, and all three genes showed a negative correlation with 36:6 MGDG and 36:6 DGDG (Table 1 and Supplementary Table S4). The expression levels of three of the five KCSs (β-ketone CoA synthases) were suppressed in MT in both the 2014 or 2017 data (Supplemental Table S5); however, the remaining two KCSs, KCS4 and KCS2b, showed upregulated profiles and were positively correlated with all three plastid lipids. All six fatty acid reductases (FARs) were significantly downregulated in MT, and CsFAR2, together with CER3, was also downregulated in the 2017 transcriptome data and in another glossy mutant of the “Newhall” orange named “Ganqi 3” (Table 1), which was consistent with the reduced alcohol and aldehyde levels^25^.

Fatty acid desaturase 2 (FAD2) initiates the formation of ER-derived plastid galactolipid biosynthesis^26^. We found that CsFAD2 showed a more obvious upregulation at 210 DAA, and the gene showed a positive correlation with MGDG and DGDG 36:6. It is worth noting that upregulation of the marker gene FAD2 was further confirmed by transcriptome data from 2017. Phospholipase D (PLD) and phospholipase C (PLC) gene families are involved in the conversion of desaturated PCs into PAs and DAGs. Two PLDs, including PLDα1 and PLDζ1, were upregulated in MT at the mature stage, and CsPLDα1(Cs1g12600) exhibited much higher expression and thus may play a more important role than PLDζ1 (Supplementary Table S5). Among all three NPCs identified, CsPLC3 showed a 1.1- and 0.62-FC increase at 210 DAA in the 2014 and 2017 data, respectively, possibly contributing to an increase in DAGs hydrolyzed from PCs. Furthermore, enhanced plastid galactolipid biosynthesis in MT was also supported by the upregulation of FAD5 and FAD7 in the 2-year transcriptome data, both of which are regarded as key genes influencing the formation of MGDGs, and FAD5 showed a highly positive correlation with plastid lipids.

Consistent with the increased JA level in MT, genes involved in lipoxygenation pathways were upregulated. Three of the four DAD1 orthologs (CsDALL2, CsDALL4, and CsDAD1), four lipoxygenases, two allene oxide synthases (AOSs), four oxo-phytodienoic acid reductases, and two JA carboxyl methyltransferases (JCMTs) were upregulated at the mature stage. Notably, the upregulation of most of the above-mentioned genes (12 out of 18) was further confirmed by the transcriptome data from 2017. Interestingly, we found that CsDAD1, CsDALL2, CsAOS (Cs3g24230), and JCMTa (Cs3g25140) were strongly correlated with plastid lipids. In addition, as a signaling hormone, JA at relatively high levels could induce the JA signaling pathways, and this induction could be signified by the degradation of JAZ proteins (JAZMONATE ZIM DOMAIN proteins)^19^. Indeed, we identified that three JAZ homologs, CsJAZ1(a) (Cs1g17210), CsJAZ1(b) (Cs1g17220), and CsJAZ8 (Cs2g03240), were significantly downregulated in MT (Supplementary Table S5), and interestingly, CsJAZ1(a) showed a strong negative correlation with plastid lipids.

### Expression patterns of lipid-related transporters and transcriptional factors

The ATP-binding cassette transporter G family (ABCG) contributes to the synthesis of wax, and three out of the seven ABCGs were consistently downregulated in MT in 2 analysis years. Among them, ABCG 32 is required for the biosynthesis and accumulation of cuticle lipids in both maize and *Arabidopsis*^27,28^, and ABCG15 is the closest homolog of CER5^29^, an indispensable gene for wax formation in *Arabidopsis*. Interestingly, both of the genes showed a highly negative correlation with plastid genes. Phospholipid exchange between different organelles also requires lipid transporters, and the protein TRIGALACTOSYLDIACY-L GLYCEROL 1 (TGD1) transfers PA from the ER to the chloroplast^30^. Two homologs of TGD1 were characterized, with CsTGD1 (a) being upregulated at the mature stage and showing high correlation with plastid lipids.

The precise regulation of lipid biosynthesis occurs at least partly at the transcriptional level. Wax-related TFs, such as MYB16 and ESE3, a homolog of the SHN family, were also significantly downregulated in MT. In addition to the known TFs in lipid-related pathways, a full list of transcriptional factors with significant differential expression and high correlation with plastid lipids is provided in Supplementary Table S6. Among these differentially expressed TFs, NAC072, which has relatively high expression and is involved in the COI1-JAZ-MYC2 JA signaling pathway in *Arabidopsis*^31^, was upregulated and showed a high correlation with plastid lipids.

### Subcellular location and transient overexpression of the three PLA1 homologs *N. benthamiana*

**Fig. 7:**
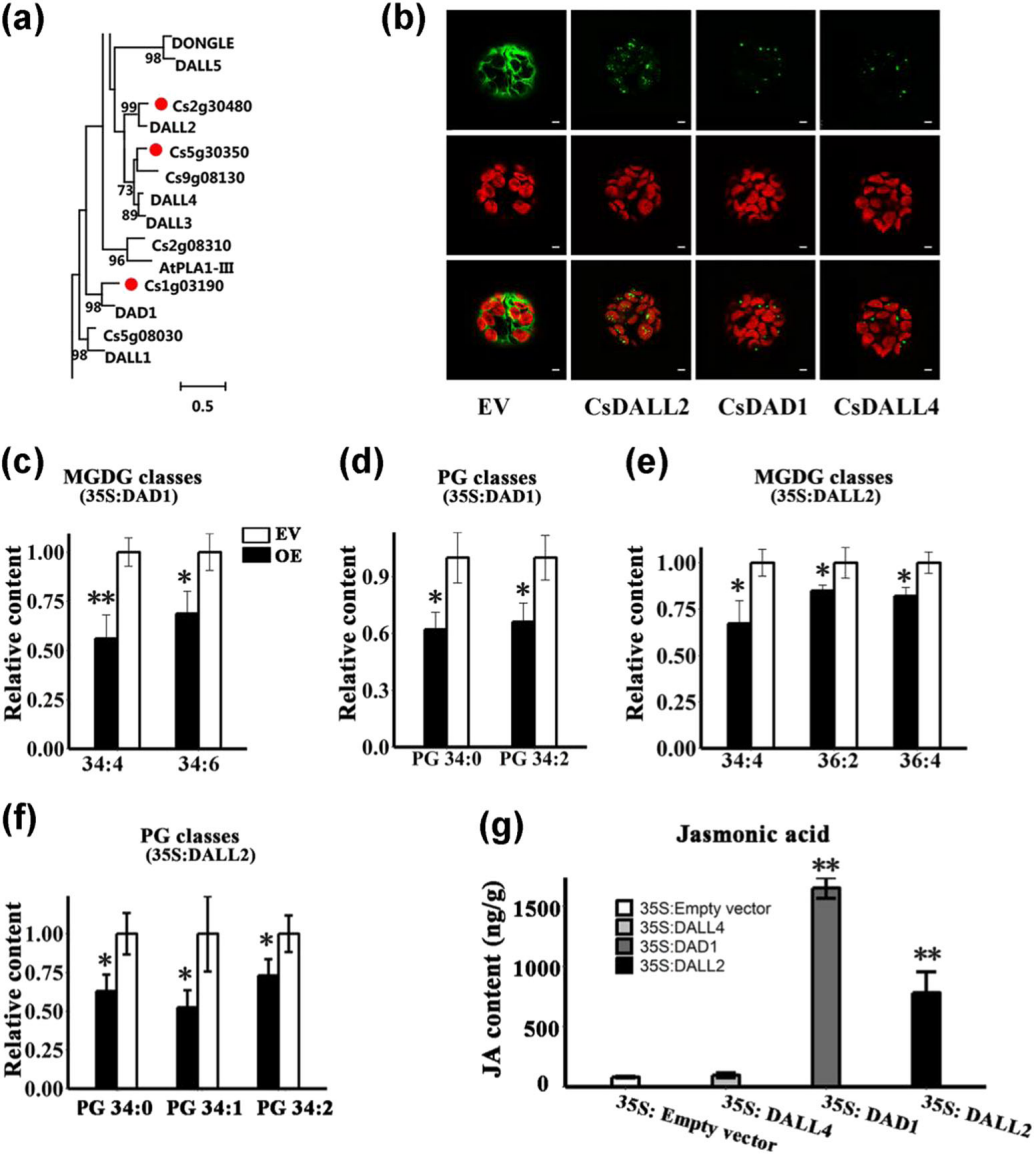
Functional characterization of DAD1-like genes in *N. benthamiana*. **a** Phylogenetic analysis of DAD1-like phospholipase genes in orange and their homologs in *Arabidopsis*. The numbers next to the branch represent the bootstrap scores (1000 replicates). The scale bar indicates the evolutionary distances computed using the JTT matrix-based method. More detailed information is available in Supplementary Fig. S7. **b** Subcellular localization of the three DAD1-like genes CsDALL4 (Cs5g30350), CsDAD1 (Cs1g03190), and CsDALL2 (Cs2g30480) in protoplasts of *N. benthamiana*. Green and red signals represent GFP and chlorophyll autofluorescence, respectively. Scale bar: 3 μm. **c–f** Relative contents of significantly changed phospholipid classes in overexpression lines of the three DAD1-like genes compared with EV. **c**, **d** Relative contents of significantly changed MGDG and PG classes observed in overexpression lines of CsDAD1. **e**, **f** Relative contents of significantly changed MGDG and PG classes observed in overexpression lines of CsDALL2. Corrected areas of each lipid subclass in replicates were summed and normalized by the average number of EV controls. Bars are the average of three normalized biological replicates ± standard deviation (SD, *n* = 3). An asterisk (*) indicates a *t*-test *p* value ≤ 0.05 and double asterisks (**) indicate a *t-*test *p* value ≤ 0.01. OE overexpression lines, EV empty vector, PG phosphatidylglycerol, MGDG monogalactosyldiacylglycerol. **g** Content of JA in overexpression lines of the three DAD1-like genes and EV. Bars are the average of three biological replicates ± standard deviation (SD, *n* = 3). JA Jasmonic acid

JA biosynthesis is closely related to plastid lipids. To determine the key lipases involved in plastid lipid degradation and JA formation, we performed a protein sequence BLAST search against phospholipase A family genes reported in *Arabidopsis*^16,20,32^. A total of 37 putative lipases were identified (Supplementary Table S7). Phylogenetic analysis of these genes revealed that three PLA1 genes were grouped within the DAD1-like gene family (Fig. 7a and Supplementary Fig. S7a). Among them, the two genes CsDAD1 and CsDALL2 showed a positive correlation with plastid lipids (Table 1). We further investigated the subcellular location of the three significantly upregulated DAD1-like genes and observed that all of them were localized in the chloroplast (Fig. 7b), which is consistent with their putative functions in liberating plastid FAs from chloroplasts. To experimentally verify the function of these genes as chloroplast lipases, we performed transient overexpression of the three lipases in *Nicotiana benthamiana*, which has been proven to be an efficient method for the functional characterization of lipid-related genes^33,34^. Relative increases over 30-fold were observed in CsDAD1, CsDALL2, and CsDALL4 overexpression lines (OE) after infiltration (Supplementary Fig. S8). Among all the chloroplast lipid classes (proportion over 0.5%) in CsDALL4 OE lines, only the content of DGDG 36:2 decreased. However, in the CsDAD1 OE lines, a number of 34-carbon chloroplast lipids were significantly (*t*-test *p* value ≤ 0.05, *n* = 3) hydrolyzed, especially 34:2 PG, accounting for approximately 33% of all PGs and 34:6 MGDG, accounting for ~27% of all MGDGs, which were significantly decreased by 33.84% and 31.26%, respectively (Fig. 7c, d and Supplementary Table S8). In the plastid lipids characterized in CsDALL2 OE lines, we observed a significant decrease in the levels of both 36- and 34-carbon lipids. For example, two 34-carbon PGs, 34:1, 34:2 (accounting for 2.6% of all PGs) and 36:4.

MGDG (accounting for ~2.0% of all MGDGs) were significantly decreased by 47.4%, 26.9% and 17.9%, respectively (Fig. 7e, f). To verify whether the hydrolyzed FAs contributed to JA formation, we tested the levels of JA in the three overexpression lines. The level of JA in EV lines was similar to that previously reported in tobacco^35^, and the CsDAll4 OE lines had almost equal amounts of JA (96.6 vs 81.4 ng/g in CsDALL4 OE and EV, respectively), indicating that the gene exerts little influence on JA biosynthesis. However, we observed 19- and 8-fold increases in JA levels in the CsDAD1 and CsDALL2 overexpression lines, respectively, suggesting that the two genes have a large influence on JA formation (Fig. 7g).

## Discussion

### Reallocation of carbon flux from cuticular wax into plastid membrane lipids in MT

Previously, He et al.^4^ reported that a decreased wax content at the mature stage results in the glossy surface of the mutant. Our research further demonstrates that wax biosynthesis in MT had already been inhibited at the fruit expansion stage, and the inhibition reached the highest level in the mature stage. Conversely, lipidomic data acquired from two independent years (2014 and 2017) demonstrated more dramatic increases in lipids in the mature stage than in the immature stage (Fig. 2b). The opposite accumulation trend of wax and plastid lipids in MT was also verified at the transcriptional level (Supplementary Table S5). These results suggest that the reduction in wax and the increase in chloroplast lipids occur simultaneously during fruit development. One possible reason for these alterations is the competition for FA substrates. FAs translocated from the plastid to the ER could either be used for the biosynthesis of various aliphatic wax components or be used for the biosynthesis of plastid-associated membranes^36^. The competition could be well-reflected by the divergent regulation profiles of KCS gene families, which function to generate VLCFA precursors for the formation of various lipids (Supplementary Table S5). Similar carbon alterations were observed in the tomato cuticular mutant named cutin deficient 2 (*cd2*). In this mutant, cuticular wax components, including alkanes and aldehydes, were reduced, and most galactolipids, together with some phospholipids, such as 34:3, 36:5 DAG, and 34:2 PG, were increased^37^. In addition, simultaneous alterations of cuticle compounds and plastid lipids were also found in many mutants, such as the mutants of *acp3, acp4* (acyl carrier protein 3, acyl carrier protein 4), *acbp3* (acyl-CoA-binding protein 3), *lacs2, lacs3, lacs4*, and *fad7*, which showed simultaneous reduction of MGDG/DGDG lipids and wax components^38–40^. However, a tighter connection was observed between the biosynthesis of wax and eukaryotic plastid lipids. As reported in *acbp3*, only eukaryotic-derived DGDGs and not prokaryotic-derived DGDGs were significantly decreased together with severely suppressed wax biosynthesis^39,41^.

As mentioned above, more carbon flux into plastid lipids could have an influence on the energy capture ability of plants, as indicated by the significantly enriched photosynthesis pathways in KEGG (Fig. 5b). Moreover, the possibly altered photosynthesis may also be reflected by the upregulation of all four members of the pyruvate kinase (PK) gene family in MT, which are involved in the transformation of photosynthetic carbon precursors for FA synthesis. The four genes all showed a high correlation with plastid lipids (Table 1). Thus, the enhanced photosynthetic ability, together with the increase in unsaturated fatty acid species in MT, may greatly contribute to the resistance of MT to disease and stress. Such a complementary carbon flux between wax and chloroplast lipids may facilitate a more effective adaptation of the plant to the environment.

### Key factors affecting carbon distribution between wax and chloroplast lipids in MT

Wax transporters may be involved in the altered carbon distribution between wax and plastid lipids, as we characterized two ABCG transporters, CsABCG15 and CsABCG32, which were downregulated in MT and negatively correlated with plastid lipids (Table 1). AtABCG15 shows the highest sequence similarity to the wax transporter CER5, and a 55% reduction in wax levels was observed in the stem of the *cer5* mutant of *Arabidopsis*^42^. CsABCG15 was found to be significantly downregulated in two glossy mutants of the “Newhall” navel orange, indicating a pivotal role of the gene in wax formation. Transcription factors may also play a role in the altered carbon distribution. WRI4 was reported to regulate wax formation through activation of LACS1, KCR, etc.^43^. Here, we found significant downregulation of WRI4 and LACS1 in MT, and both genes synergistically negatively correlated with plastid lipids. However, we did not find sequence differences of these genes between WT and MT; thus, these processes were unlikely to be the cause of the alteration. Lipid genes were also involved in this process. CsFAD2 showed upregulation in MT and a positive correlation with the two main 36:6 galactolipids, which demonstrated a clear activation of the eukaryotic pathway for galactolipid formation in MT. In addition, the expression level of FAD2 was found to be positively correlated with the eukaryotic pathway in suboptimal temperatures either in the “16:3 plant” *Arabidopsis* or in the “18:3 plant” wheat^44^. The increase in fully desaturated 36:6-carbon MGDG and DGDG at the mature stage in MT could have resulted from the increase in FAD7 expression. Mutants of *fad7-1* and its closely related homolog *fad8* exhibited decreased plastid lipid levels, which further resulted in low JA levels due to the lack of lipid precursors^45,46^.

FATB prefers saturated acyl-ACP as the substrate to release free FAs to the ER, and decreased wax crystals were found in *fatb* in *Arabidopsis*^47^. We also found that CsFATB was downregulated in two glossy mutants of the “Newhall” navel orange (Table 1). Interestingly, the gene also showed a highly negative correlation with the three main plastid lipids, which may be the result of elevated FA export from chloroplasts, as 40% and 55% increases in FA synthesis and oleate acid export were found in *fatb*, respectively, in an attempt to maintain lipid homeostasis^10^. However, how these increased FAs were assimilated into PCs for eukaryotic plastid lipid biosynthesis remains unknown. The two upregulated GPATs (acyl-CoA: glycerol-3-phosphate acyltransferase) that putatively initiate the de novo synthesis of the PA-DAG-PC pathway were expressed at much lower levels and thus may be insufficient for the biosynthesis of bulk amounts of PC. Further analysis showed that all members in the LPAAT (acyl-CoA: lysophosphatidic acid acyltransferase) gene family were downregulated, which may be explained by the fact that most lysophospholipid acyltransferases have forward and reverse functions or that the transcriptome variation may be inconsistent with the lipidomic data due to the involvement of some important posttranscriptional processes. Further studies are needed to clarify how fatty acids are used for the biosynthesis of PCs.

### Initiation of jasmonate synthesis in the “Newhall” navel orange

Lipid degradation is indispensable in the maintenance of lipid homeostasis and lipid-mediated signaling^48^. Sequence comparison and phylogenetic analyses of PLA lipases identified in citrus and *Arabidopsis* showed that all three upregulated lipases were clustered into the DAD1-like gene subgroup. The chloroplast subcellular location of the three genes suggested their roles as chloroplast-localized lipases (Fig. 7b), which is consistent with previous reports in *Arabidopsis* that all DAD1-like gene members are located in the chloroplast^32^. We found that both plastid lipid classes, including galactolipids (MGDGs and DGDGs) and phospholipids (PGs), were significantly reduced in CsDAD1 and CsDALL2 overexpression lines, which is consistent with the results of in vitro studies that AtDAD1 and AtDALL2 could hydrolyze both phospholipids and galactolipids^32,49^. Furthermore, our results showed a 34-carbon substrate preference of CsDAD1, as indicated by the significant decrease in 34:0 and 34:2 PGs and highly unsaturated galacotolipids, such as 34:4 and 34:6 MGDGs (Fig. 7c, d). AtDALL2 showed the strongest activity toward MGDG in vitro^32^, and we did find that 34:4, 36:4, and 36:2 MGDGs were decreased in OE lines. Furthermore, we verified that this gene may also hydrolyze PGs, as indicated by the significant decrease in three PG subclasses in the OE lines (Fig. 7f). Consistent with the dramatic degradation of plastid lipids, we observed significantly elevated levels of JA in CsDAD1 and CsDALL2 overexpression lines, and in the CsDALL4 OE lines with few degraded lipids, we observed almost equal amounts of JA compared with that in the EV. Thus, the functional characterization results of the three genes were in accordance with our plastid-gene correlation prediction that CsDALL4 showed no high correlation with plastid lipids and possessed less plastid lipid hydrolyzation ability.

Based on the characterized function of the upregulated lipases in this study, we hypothesized that the elevated JA levels in MT may result from the enhanced lipid degradation pathways catalyzed by lipases to maintain chloroplast homeostasis, which is of great importance for the integrity of chloroplasts^15^. Given that CsDAD1 and CsDALL2 both presented little preference for 36:6 MGDG and DGDG, which account for over 60% of the respective lipid classes and displayed elevated levels in MT (Supplementary Table S8), there should be other chloroplast-localized lipases involved in the process. These lipases could be characterized in future work to fully identify the key lipase genes that initiate JA synthesis in “Newhall” navel oranges.

## Materials and methods

Fruits of the mutant (MT) and wild type (WT) were harvested in three adjacent orchards (one contained mutant trees, and the other two contained the grafted branches from the MT) in Anyuan County of Jiangxi Province in China in 2014 and 2017 with similar management conditions. Three biological replicates of MT and WT samples were collected in the preharvest stage at 90 DAA, 150 DAA, and 210 DAA, with each replicate containing at least ten representative fruits of uniform size. Flavedo tissues were rapidly sampled from each fruit, immediately immersed in liquid nitrogen, and then stored at −80 °C for transcriptomic and metabolomic analysis.

### Total wax extraction and analysis by GC-MS

The total wax was extracted according to a previously reported method^4,23^. Intact fruits were immersed in chloroform for 2 min, twice. After the addition of 200 μl (1 μg/μl) n-tetracosane as an internal standard, the extracts were dried by a gentle stream of nitrogen (N_2_) and then derivatized in pyridine for 30 min at 50 °C, followed by treatment with bis-N,N-(trimethylsilyl) trifluoroacetamide (BSTFA) containing 1% trimethylchlorosilane (Sigma) for 40 min at 60 °C. After derivatization, the mixture was concentrated with a gentle nitrogen gas flow and redissolved by chloroform. GC-MS (Thermo Fisher, ISQII, USA) and an Agilent DB-1 column (30 m × 25 μm i.d. × 0.1 μm) were used to perform wax analysis. The instrument parameter settings were consistent with those of previous reports^23^. The contents of all acquired compounds were calculated by comparison with the internal standard. Three biological replicates at 90 DAA, 150 DAA, and 210 DAA were analyzed.

### Lipid extraction and analysis using UPLC-MS/MS

Lipids were extracted from fruits of the WT and MT collected at 150 DAA and 210 DAA using the protocol described below: Briefly, 30 mg of freeze-dried flavedo tissue was ground into a fine powder. Metabolites from each aliquot were extracted with 1 ml of precooled (−20 °C) extraction buffer (homogenous methanol/methyl-*tert*-butyl-ether [1:3] mixture). After 10 min of incubation at 4 °C and sonication for 10 min in an ultrasonication bath, 500 μl of a water/methanol mixture was added. Samples were then centrifuged (5 min, 20,817 *g*), which led to the formation of two phases: a lipophilic phase and a polar phase. A lipophilic phase of 500 ml was collected and dried under vacuum. The lipophilic phase was resuspended in 200 μl of isopropanol and used for lipid analysis.

Lipid analysis was performed on a 5600 plus Accurate-Mass Q-TOF (AB SCIEX 5600 plus Q-TOF, USA) mass spectrometer system. Liquid chromatography was carried out using a UPLC with an autosampler (Shimadzu Corporation, Kyoto, Japan). An ODS C-18 (Shimadzu Corporation) column (2.1 mm × 150 mm, 2 μm) was used at 40 °C. The injected volume of the sample was 10 μL. The elution gradient was carried out with a binary solvent system consisting of 10 mM ammonium acetate in 30% methanol in water (solvent A) and 10 mM ammonium acetate in 10% acetonitrile in isopropanol (solvent B) at a constant flow rate of 250 μl/min. A linear gradient profile with the following proportions (v/v) of solvent B was applied: 0–2 min, 25% B; 2–5 min, 25–40% B; 4–22 min, 40–95% B, 22–27 min, 95% B; 27–27.5 min, 100–25% B; and 27.5–32 min, 4.5 min for re-equilibration at 25% B. The MS spectra were processed with Analyst TF 1.7 (SCIEX, USA). MS conditions were as follows: source voltage, 5.5 kV; source temperature, 550 °C; declustering potential, 100 V; gas 1 and 2 (nitrogen), 50 psi; and curtain gas (nitrogen), 35 psi. A data-dependent analysis was conducted on the 15 most abundant metabolite ions in a full-scan cycle (100 ms). The scan ranges of *m*/*z* of precursor ion and product ion were set as 400–1000 Da and 80–1000 Da, respectively. The selected precursor ions were >4000 cps with a charge state of 1 at an MS tolerance of 50 mDa. The collision energy of the fragment in the collision cell was set at 30 ± 10 eV. Compounds were identified based on their accurate mass for which tolerance was set to a 5 ppm mass window and the MS2 fragmentation pattern of the precursor ion. The data were obtained using Analyst TF 1.6 and MultiQuantTM and processed using PeakView 2.0 software. Accurate molecular weight and fragmentation ion information were acquired using LipidView 2.0 to generate an in-house dataset. The identification of glycerolipid classes and species was based on retention time, accurate *m*/*z*, and fragmentation ion patterns according to a previous report^50^.

### Measurement of JA phytohormones in “Newhall” navel orange and *N*. *benthamiana*

Measurement of JA was performed according to a previously reported method (He et al.^4^). One hundred milligrams of each samples was ground and mixed with 800 μl of solvent containing methanol/H_2_O/acetic acid (80:19:1, v/v/v) and internal standards for JA determination (DHJA; Olomouc, Czech Republic). The analysis of hormone extracts was performed using an Agilent 1100 HPLC system coupled to an Agilent API3000 mass spectrometer. Identification and annotation of JA were conducted according to a reported method^51^. Three biological replicates of “Newhall” navel oranges (WT and MT) and *N*. *benthamiana* (overexpression lines and control lines) were utilized. Flavedo and juice sac tissues in “Newhall” navel oranges and infiltrated leaves of *N. benthamiana* were used.

### Flavedo RNA-seq analysis and qRT-PCR validation

Flavedo tissues sampled from WT and MT at 150 DAA and 210 DAA were used for RNA extraction with the method reported by Liu et al.^52^. Stranded mRNA-Seq libraries were constructed using the KAPA Stranded mRNA-seq kit (Kapa Biosystems, Cape Town, SA) following the manufacturer’s recommendations and were sequenced with the Illumina HiSeq X Ten system (paired-end 150-bp reads) with three biological replicates for 150 DAA, two for 210 DAA in 2014 and three for 210 DAA in 2017. Approximately eight million clean reads were mapped to the reference genome from *Citrus sinensis*^53^ using Tophat (with the G parameter). The uniquely mapped reads were extracted for estimating the expression levels of protein-coding genes using Cufflinks, and FPKM was used as the unit of measurement to estimate transcript abundance. Annotation of genes was based on the data acquired from the *C. sinensis* annotation website: http://citrus.hzau.edu.cn/orange/index.php. For DEG analysis, a count table was first generated by featureCounts. DEGs were identified by applying statistical tests to the WT and MT groups in the same stage, which was performed using DESeq2 in the R statistical package. All genes with a Benjamini–Hochberg adjusted *p* value smaller than 0.05 and with a FC larger than 1.5 were reported as DEGs.

A total of 15 genes putatively involved in lipid and wax pathways were used to validate the RNA-seq results through quantitative real-time polymerase chain reaction (qRT-PCR), and the results are presented in Supplementary Figs. S9 and S10. Real-time qRT-PCR was performed on the Roche LightCycler 480II System (Roche Diagnostics). The acquired data were processed with the 2^−ΔΔCt^ analysis method^54^.

Protein sequences of DEGs were extracted and submitted to KOBAS3.0 for KEGG enrichment analysis^55^. Protein sequences of genes that showed a high positive correlation with the three plastid lipids were extracted and submitted to the PlantTFDB 4.0 database (http://planttfdb.cbi.pku.edu.cn/index.php) for GO enrichment analysis.

### Analysis of lipid-related genes in the “Newhall” navel orange and annotation of transcription factors

To identify lipid-related genes in the “Newhall” navel orange, the protein sequences of lipid-related genes in *Arabidopsis* were used to run the BLASTp program (Blast + version 2.3.0) against protein sequences of all up/downregulated genes in the “Newhall” navel orange, with a threshold of an *E*-value < e^−6^ and an identity ≥30%. The extracted sequences were further confirmed with conserved domains using HMMER (v3.1b2) (http://hmmer.org/). The results are shown in Supplementary Table S5.

### Phylogenetic analysis

Multiple sequence alignment was performed using the MUSCLE program based on amino acid sequences of phospholipase A family genes in *Arabidopsis* and Citrus and then manually adjusted in MEGA (version 7.0.14). The maximum likelihood phylogenetic tree was constructed using MEGA (version 7.0.14) with the JTT model and 1000 bootstrap resamplings. Bootstrap values above 70 are shown. Gene IDs used to generate phylogenetic trees are provided in Supplementary Table S7, and the results of the phylogenetic tree are shown in Supplementary Fig. S7.

### Subcellular localization of CsDAD1, CsDALL2, and CsDALL4

The 35S:CsDAD1–GFP, 35S:CsDALL2–GFP, and 35S:CsDALL4–GFP fusion constructs were produced by inserting the coding sequence without a stop codon into pH7LIC6.0 (35S-GFP). The GFP fusion constructs were introduced into protoplasts prepared from *N. benthamiana* rosette leaves by polyethylene glycol-mediated transformation^56^. Protoplasts were incubated at 24 °C in the dark for 18 h. The florescence images were captured using a confocal laser-scanning microscope (TCS SP2; Leica, Wetzlar, Germany). GFP signals were imaged with excitation at 488 nm, and the emission signal was collected between 500 and 555 nm. The autofluorescence signal of chlorophyll was collected between 650 and 750 nm. Details of primers for constructs are listed in Supplementary Table S9.

### Transient overexpression of CsDAD1, CsDALL2, and CsDALL4 in *N. benthamiana*

Transient expression in *N. benthamiana* leaves was performed as previously reported^57,58^ with some minor modifications. The full-length cDNA of the three genes was amplified and cloned into the Gateway Entry vector pDNOR 221, which was then recombined with the resulting plasmids PK7WG2D. Two *Agrobacterium* tumefaciens GV3101 strains harboring either the constructed vector or the gene coding for the p19 viral suppressor protein were mixed together so that the final OD 600 of each culture was equal to 0.2 prior to infiltration. Infiltration was performed on the underside of leaves of 1-month-old *N. benthamiana* that had been cultured in a 24 °C plant growth room with a 10:14 dark:light cycle. Infiltration experiments were conducted in triplicate, and each replicate contained nine leaves harvested from three plants with uniform size. After infiltration, *N*. *benthamiana* plants were grown for another 5 days before leaf discs were harvested, pooled and stored at −80 °C. The method used for *N. benthamiana* lipid analysis was the same as described above. The primers used are listed in Supplementary Table S9.

## Supplementary information

Supplementary Figures S1-10

Relative levels of lipids at two stages in 2014

Relative levels of lipids at mature stage in 2017

Supplementary Table S3. Significantly correlated lipid compounds

Supplementary Table S4. Genes tht showed significant correlation with phosphalipid species (except TAG)

Supplementary Table S5. Expression of putative lipid genes identified in citrus

Differently expressed transcription factors that show high correlation with two main kinds of plastidlipids

Supplementary Table S7. Phosphalipase A family genes characterized in citrus

Supplementary Table S8. Relative levels of plastid lipids in overexpression and EV lines in N. benthamiana

Primers used in this study
