# Supplementary material for: Lipidomic and transcriptomic analysis reveals reallocation of carbon flux from cuticular wax into plastid membrane lipids in a glossy “Newhall” navel orange mutant
Source: Hortic Res. 2020 Apr 1;7:41. doi: 10.1038/s41438-020-0262-z (PMC7109130; doi:10.1038/s41438-020-0262-z)
Supplement: Supplementary file 1 — Supplementary Figures S1-10 [file 41438_2020_262_MOESM1_ESM.pdf]

# Supplementary Figures

## Lipidomic and transcriptomic analysis reveals re-allocation of carbon flux from cuticular wax into plastid membrane lipids in a glossy 'Newhall' navel orange mutant

Haoliang Wan[1#], Hongbo Liu[2#], Jingyu Zhang[1], Yi Lyu[3], Zhuoran Li[1], Yizhong He[1], Xiaoliang Zhang[1], Xiuxin Deng[1], Yariv Brotman[4], Alisdair R. Fernie[5], Yunjiang Cheng[1*], Weiwei Wen[1*]

[1]Key Laboratory of Horticultural Plant Biology (MOE), College of Horticulture and Forestry Sciences, Huazhong Agricultural University, Wuhan, 430070 China

[2]National Key Laboratory of Crop Genetic Improvement, Huazhong Agricultural University, Wuhan 430070, China

[3]Key Laboratory for Space Bioscience and Biotechnology, School of Life Sciences, Northwestern Polytechnical University, Youyi Xilu 127, 710072, Xi'an, Shaanxi, China

[4]Department of Life Sciences, Ben-Gurion University of the Negev, Beersheba, Israel

[5]Max-Planck-Institute of Molecular Plant Physiology, Am Muehlenberg 1, Potsdam-Golm 14476, Germany

# contribute equally

Address correspondence to wwwen@mail.hzau.edu.cn and yjcheng@mail.hzau.edu.cn

## Supplementary Fig. S 1

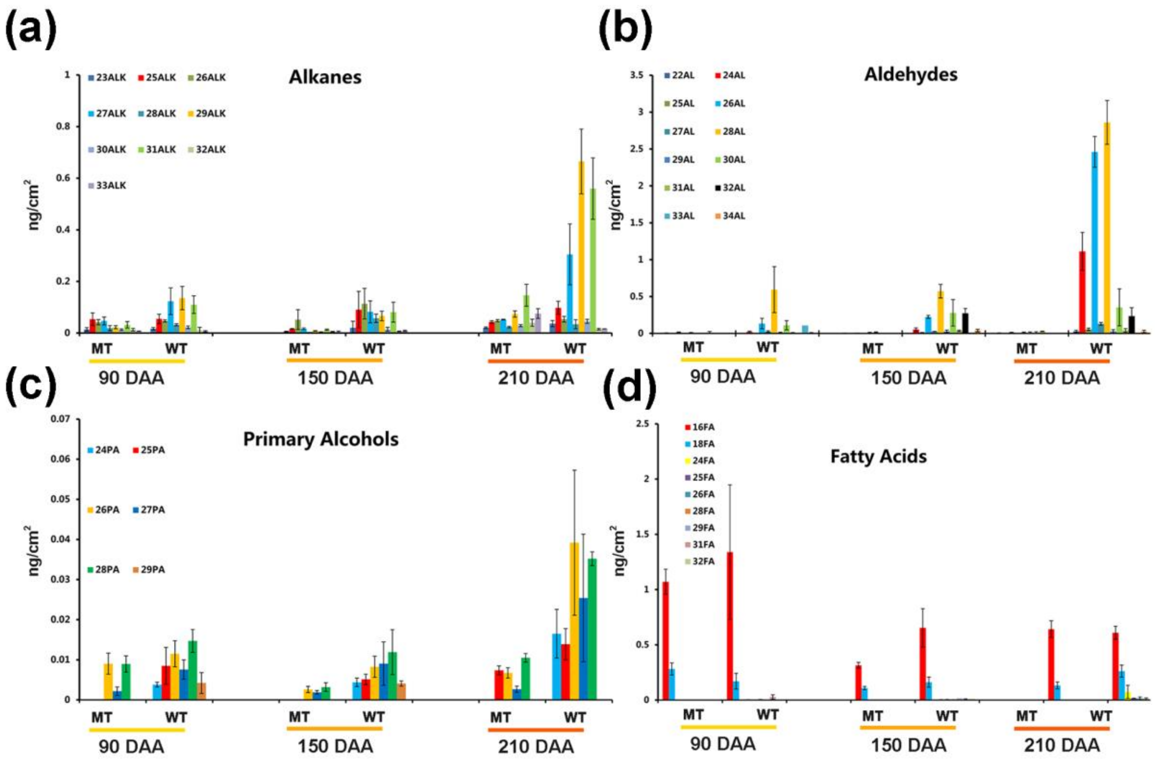


**Fig. S1. Levels of wax subclass components in MT and WT flavedo at the three stages**. (a) Levels of Alkanes; (b) Levels of Aldehydes; (c) Levels of Primary alcohols; (d) Levels of Fatty acids. Values are average of three biological replicates ± standard deviation (SD) (n = 3).

**Supplementary Fig. S 2**

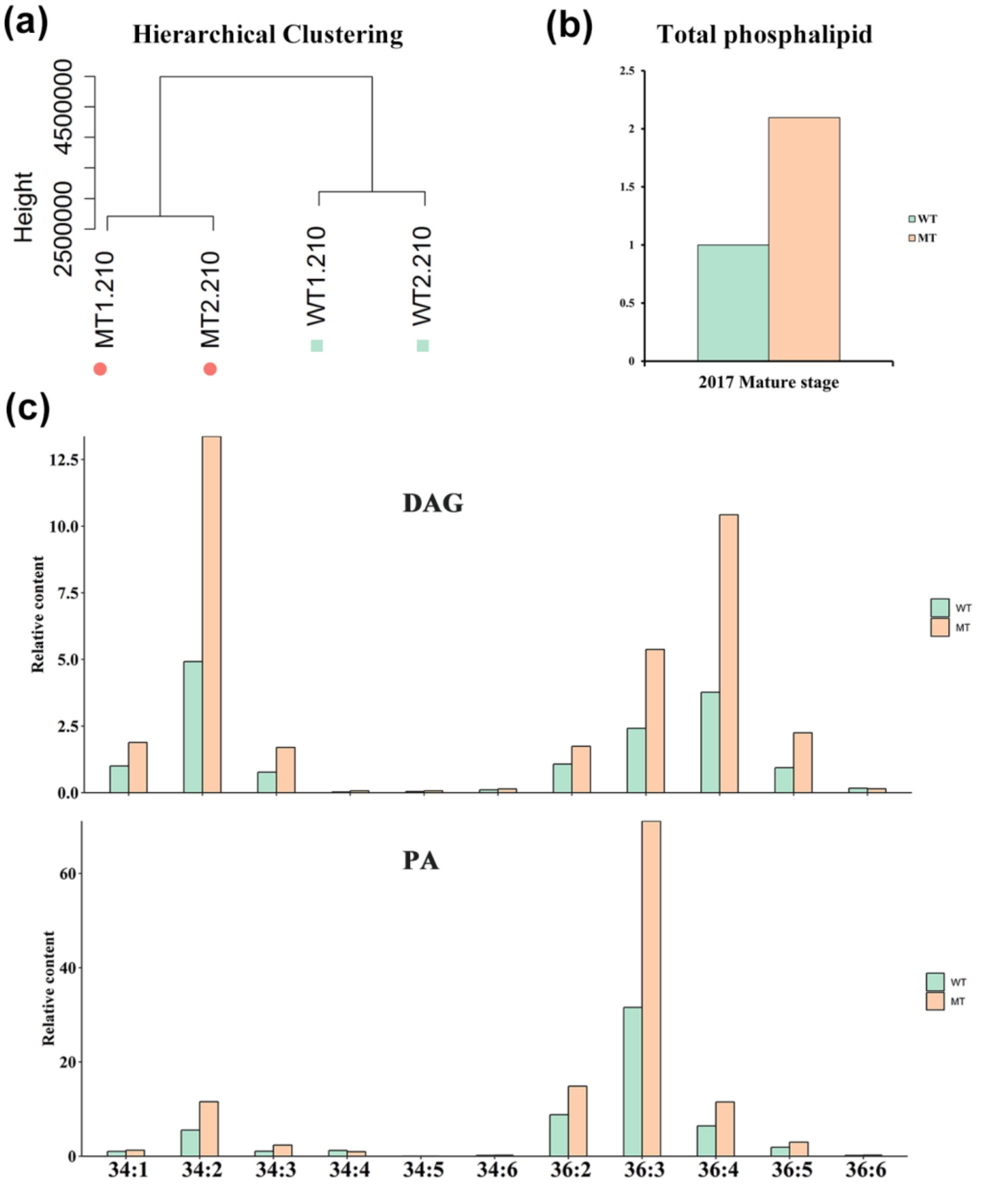


**Fig. S2. Contents of glycerolphospholipids in WT and MT on 210 DAA in year 2017.** (a) Hierarchical clustering analysis of all samples used for lipid analysis in WT and MT in 2017 based on average hierarchical clustering. Numbers indicate days after anthesis; WT, wild type; MT, mutant type. (b) Relative content of total phospholipids observed on 210 DAA in the year of 2017. Corrected areas of all phospholipid classes in each replicate were summed and normalized by the average number of WT. Bars are the average of two normalized biological replicates. (c) Relative contents of two main lipid precursors PA and DAG on 210 DAA. Corrected area of each subclass was summed and normalized by the average number of 34:1 carbon molecules in DAG and PA in WT, respectively. Bars are the average of two normalized biological replicates on 210 DAA. DAG, Diacylglycerol; PA, phosphatidic acid.

**Supplementary Fig. S 3**

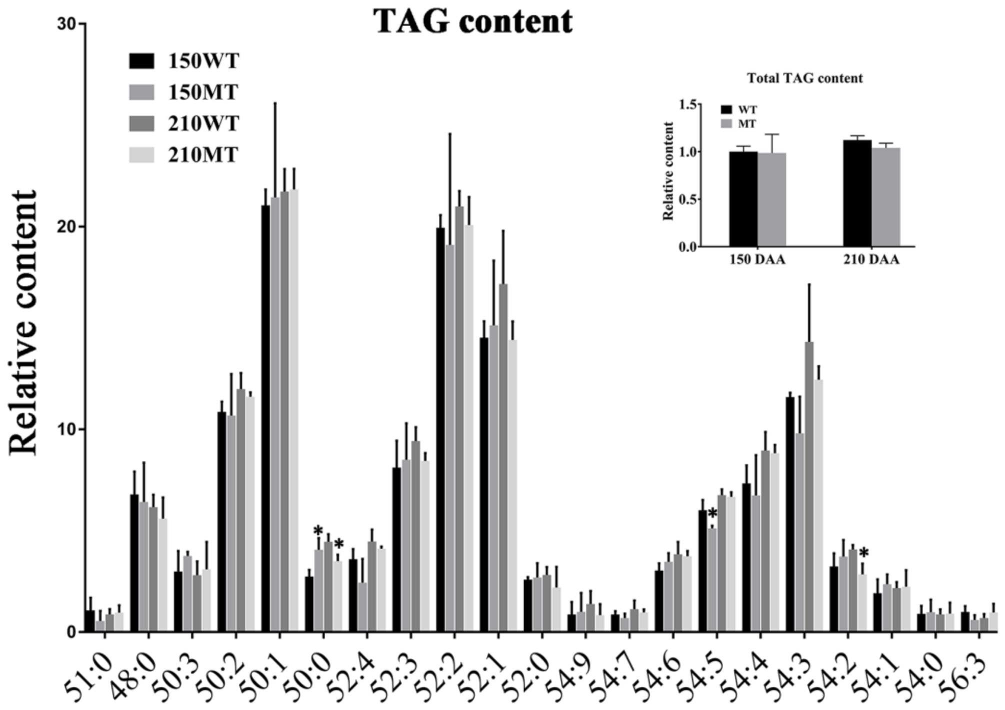


**Fig. S3. Relative contents of TAG in WT and MT on 150 and 210 DAA in 2014.** Corrected area of each TAG subclass was summed and normalized by the average number of 56:3 subclass on 150 DAA in WT, respectively. Bars are the average of three normalized biological replicates ± standard deviation (SD, n = 3). * means *t*-test p value ≤ 0.01. TAG, triacylglycerol.

## Supplementary Fig. S4

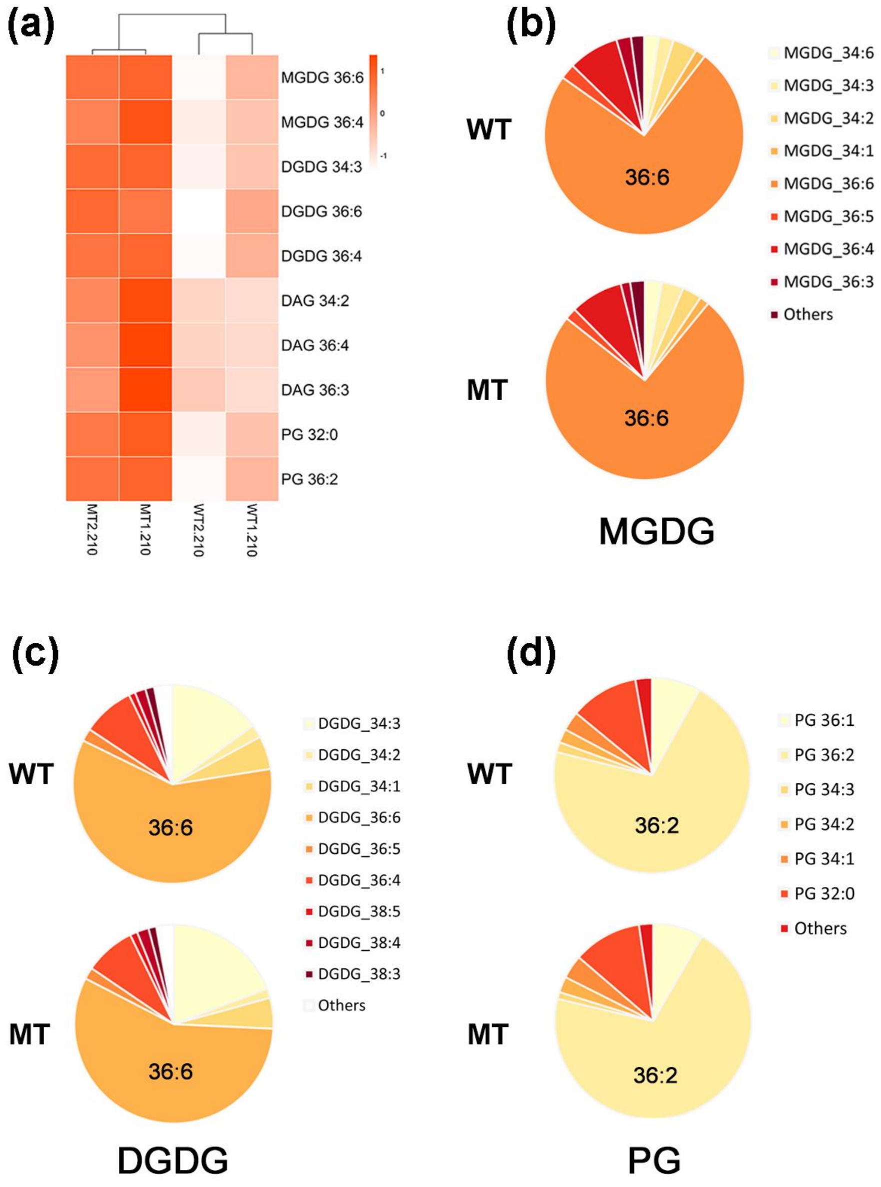


**Fig. S4. Variations of main plastid lipids on 210 DAA in 2017.** (a) Heatmap illustrating the variations of the three main plastid lipids: MGDG, DGDG and PG and their direct precursor DAG. Subclasses accounting for more than 7% in each class were used to draw the heatmap. The data were scaled by row and the clustering was based on Euclidean distance. (b) (c) (d) Proportions of subclasses in the three main plastid lipids: MGDG, DGDG, and PG. Subclasses accounting for more than 1% in each class were displayed in the diagram. MGDG, monogalactose diacylglycerol; DGDG, digalactose diacylglycerol; PG, phosphatidylglycerol.

Supplementary Fig. S5

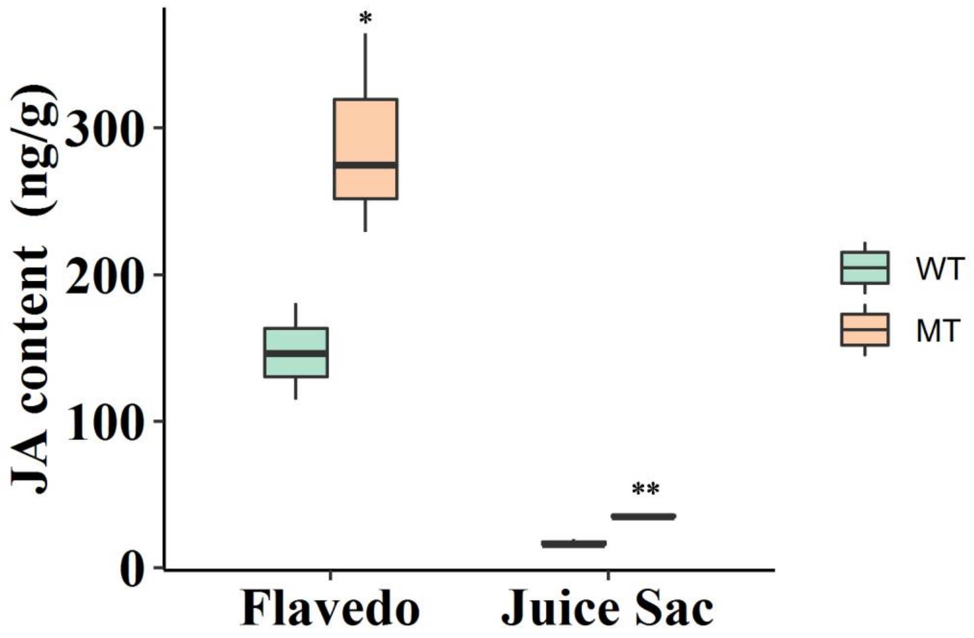


**Fig. S5. Contents of JA in flavedo and juice sac on 210 DAA.** Center lines are medians, boxes show the upper and lower quartiles (n = 3). * means *t-test* p value ≤ 0.05; ** means *t-test* p value ≤ 0.01.

## Supplementary Fig.S6

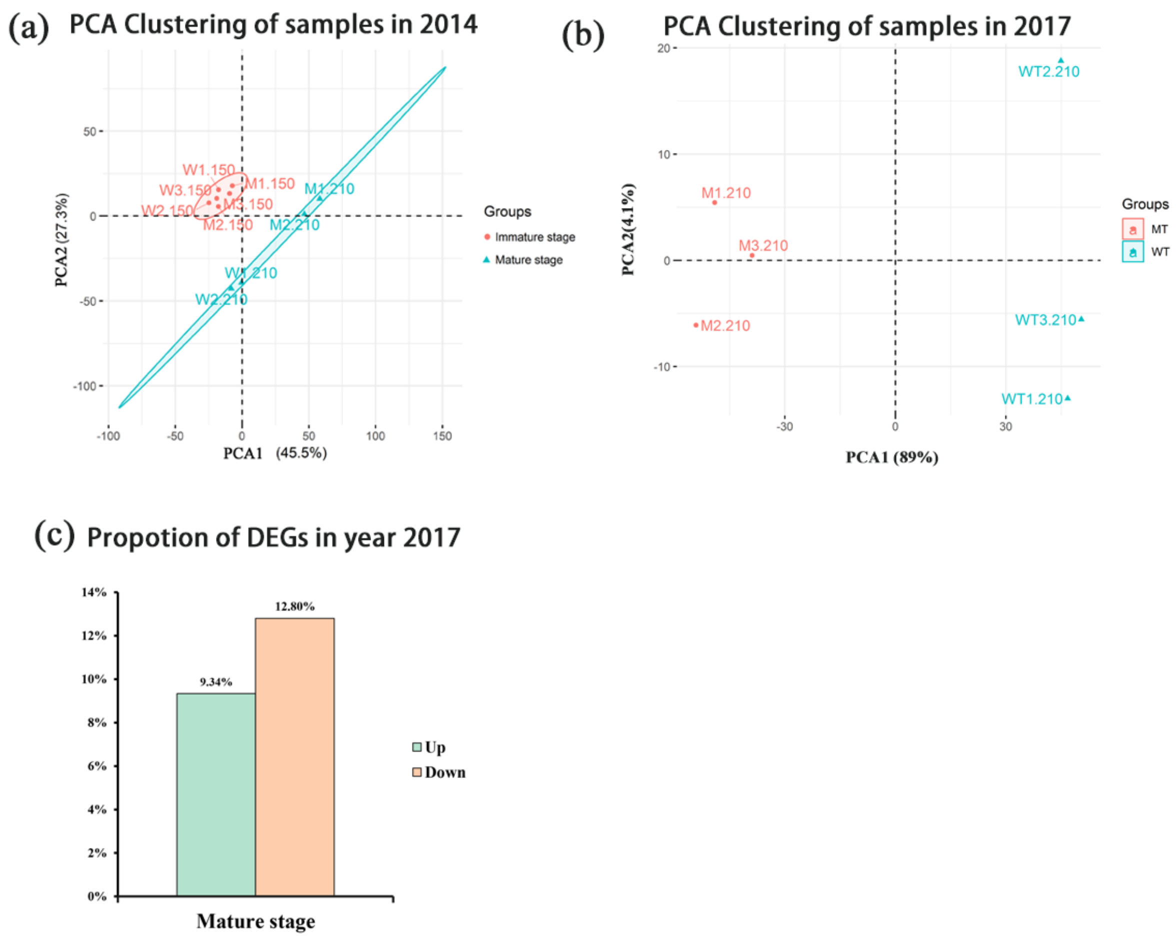


**Fig. S6. PCA clustering of all samples used for transcriptomic analysis and proportion of DEGs in year 2017. (a)** PCA clustering of smaples used in year 2014.The ellipses represent group membership assuming the 95% confidence limit. **(b)** PCA clustering of smaples used in year 2017. **(c)** Proporion of DEGs characterized in year 2017. W: wild type, M: mutant type. The number 150 or 210 means days after anthesis. Samples from immature or mature stage are indicated in red circle or cyan triangle.

**Supplementary Fig. S7**

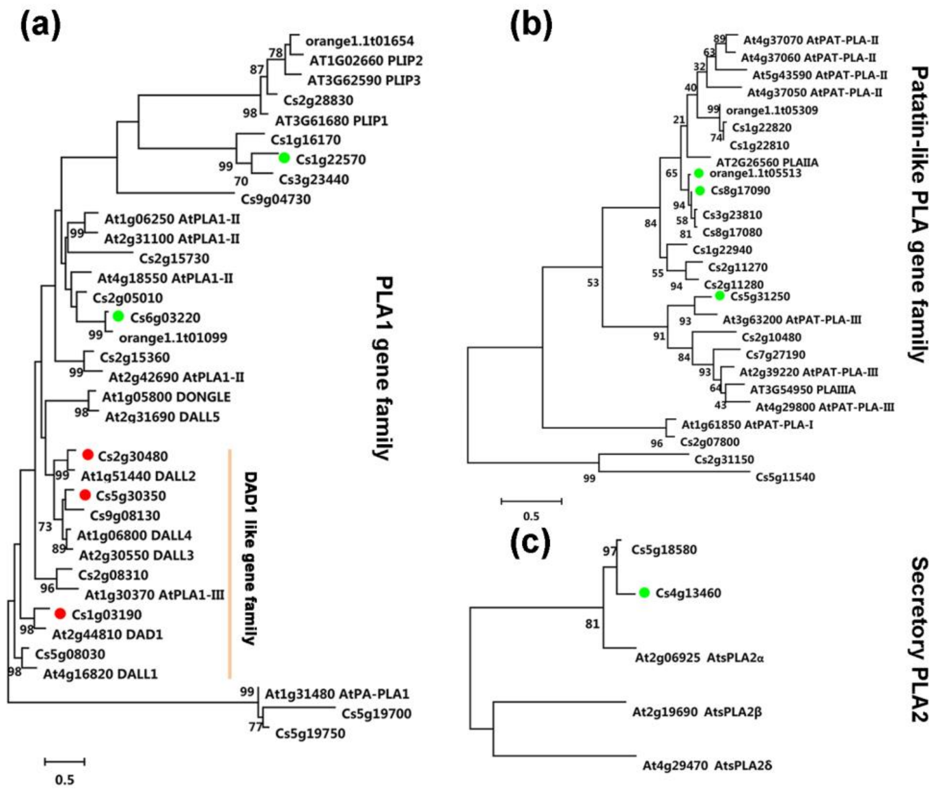


**Fig. S7. Phylogeny of phophalipase A family genes in Arabidopsis and 'Newhall' navel orange.** (a) The phylogenetic tree of PLA1gene family. (b) The phylogenetic tree of Patatin-like PLA gene family. (c) The phylogenetic tree of Secretory PLA2 gene family. The numbers next to the branch represent the bootstrap values (1000 replicates). The scale bar indicates the evolutionary distances computed using the JTT matrix-based method. Circles in red indicates that genes are significantly up-regulated in MT, and circles in green indicates that genes are significantly down-regulated in MT. Genes used for the construction of phylogenetic tree are listed in Supplementary Table S7.

Supplementary Fig. S8

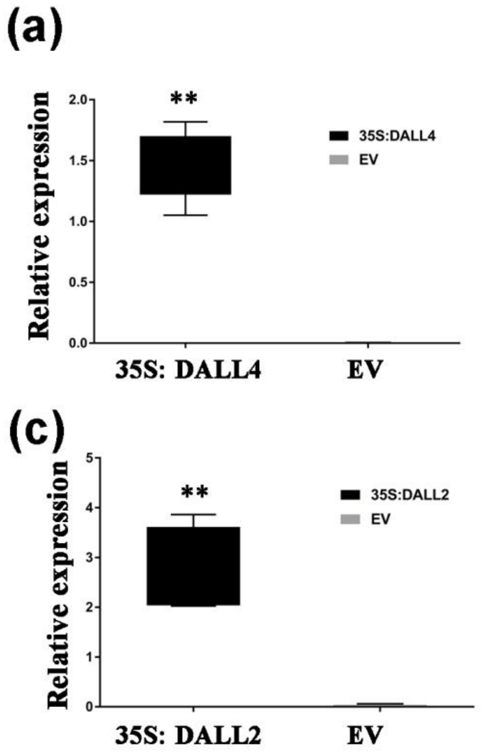


**Fig. S8. Relative expression level of the three DAD1-like genes in *N. benthamiana*.** (a) Relative expression of DALL4. (b) Relative expression of DAD1. (c) Relative expression of DALL2. Relative expression levels of the three genes were normalized by expression level of EV. The genes and primer sequences for qPCR are listed in Supplementary Table S9. ** means *t*-test p value ≤ 0.01, n = 3.

## Supplementary Figure S9

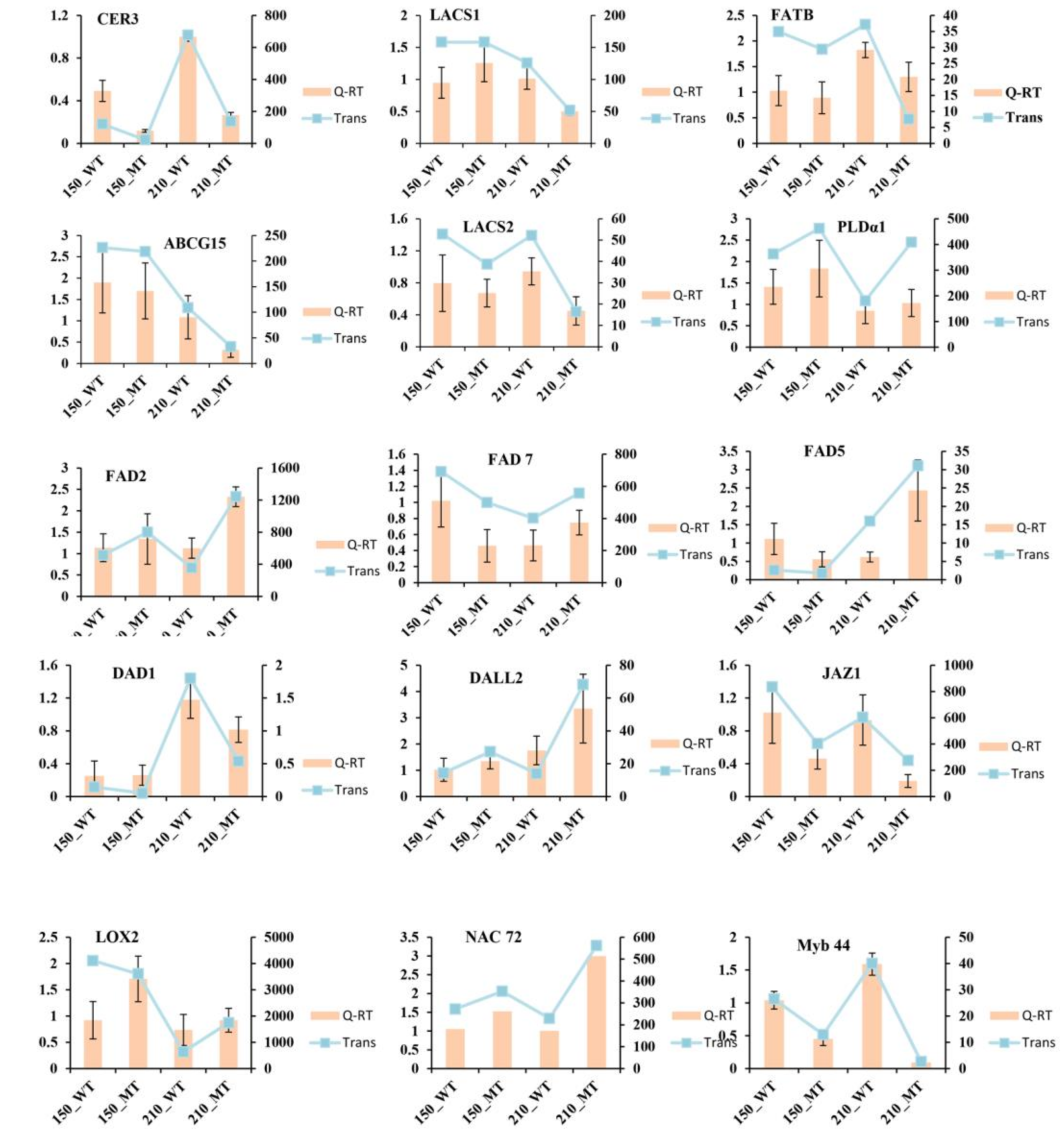


**Fig. S9. Consistency between RNA-Seq-based and qPCR-based transcript quantification in year 2014.** Fifteen genes involved in lipid-related pathways were chosen for qPCR validation. The genes and primer sequences for qPCR are listed in Supplementary Table S9. The gene expression was validated at both time points.

**Supplementary S10**

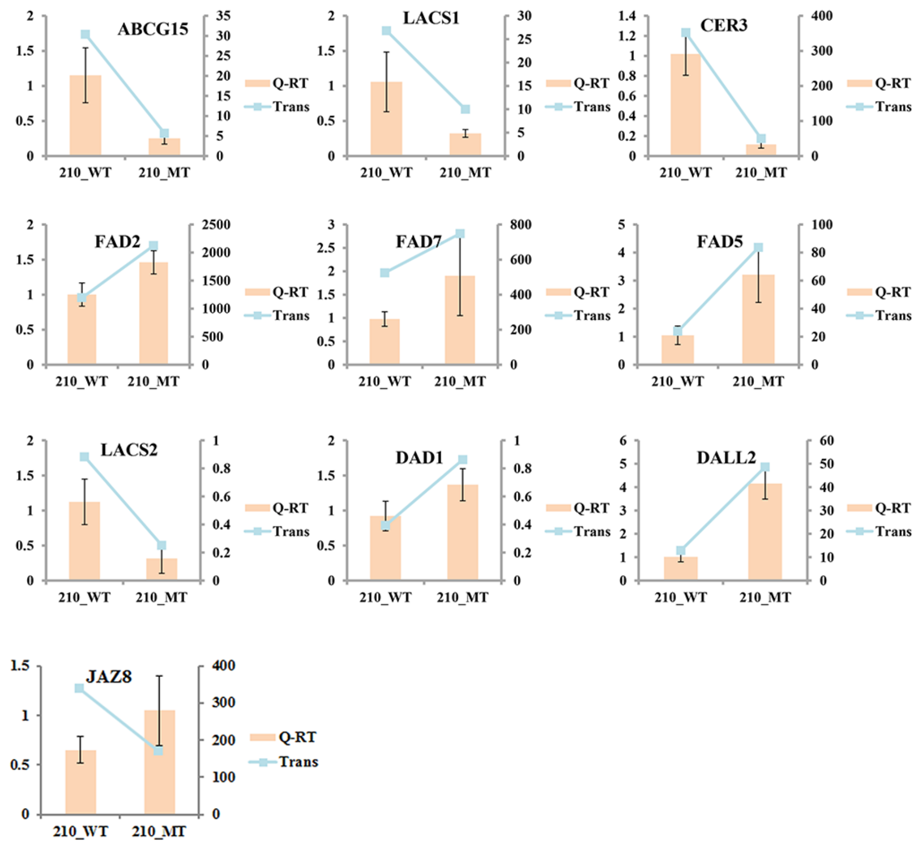


**Fig.S10. Consistency between RNA-Seq-based and qPCR-based transcript quantification in year 2017.** Ten genes involved in lipid-related pathways were chosen for qPCR validation. The genes and primer sequences for qPCR are listed in Supplementary Table S9.
